# Hybrid dysgenesis in *Drosophila virilis* results in clusters of mitotic recombination and loss-of-heterozygosity but leaves meiotic recombination unaltered

**DOI:** 10.1186/s13100-020-0205-0

**Published:** 2020-02-15

**Authors:** Lucas W. Hemmer, Guilherme B. Dias, Brittny Smith, Kelley Van Vaerenberghe, Ashley Howard, Casey M. Bergman, Justin P. Blumenstiel

**Affiliations:** 1 Department of Ecology and Evolutionary Biology, University of Kansas, Lawrence, KS 66045 USA; 2 Present Address: Department of Biology, University of Rochester, Rochester, NY 14627 USA; 3 Department of Genetics and Institute of Bioinformatics, University of Georgia, Athens, GA 30602 USA; 4 Department of Molecular Biosciences, University of Kansas, Lawrence, KS 66045 USA

**Keywords:** *Drosophila virilis*, Hybrid dysgenesis, Transposons, Meiotic recombination, Mitotic recombination

## Abstract

**Background:**

Transposable elements (TEs) are endogenous mutagens and their harmful effects are especially evident in syndromes of hybrid dysgenesis. In *Drosophila virilis*, hybrid dysgenesis is a syndrome of incomplete gonadal atrophy that occurs when males with multiple active TE families fertilize females that lack active copies of the same families. This has been demonstrated to cause the transposition of paternally inherited TE families, with gonadal atrophy driven by the death of germline stem cells. Because there are abundant, active TEs in the male inducer genome, that are not present in the female reactive genome, the *D. virilis* syndrome serves as an excellent model for understanding the effects of hybridization between individuals with asymmetric TE profiles.

**Results:**

Using the *D. virilis* syndrome of hybrid dysgenesis as a model, we sought to determine how the landscape of germline recombination is affected by parental TE asymmetry. Using a genotyping-by-sequencing approach, we generated a high-resolution genetic map of *D. virilis* and show that recombination rate and TE density are negatively correlated in this species. We then contrast recombination events in the germline of dysgenic versus non-dysgenic F1 females to show that the landscape of meiotic recombination is hardly perturbed during hybrid dysgenesis. In contrast, hybrid dysgenesis in the female germline increases transmission of chromosomes with mitotic recombination. Using a de novo PacBio assembly of the *D. virilis* inducer genome we show that clusters of mitotic recombination events in dysgenic females are associated with genomic regions with transposons implicated in hybrid dysgenesis.

**Conclusions:**

Overall, we conclude that increased mitotic recombination is likely the result of early TE activation in dysgenic progeny, but a stable landscape of meiotic recombination indicates that either transposition is ameliorated in the adult female germline or that regulation of meiotic recombination is robust to ongoing transposition. These results indicate that the effects of parental TE asymmetry on recombination are likely sensitive to the timing of transposition.

## Background

Hybridization can cause genome instability and reveal incompatibilities between parental genomes [1–3]. Transposable elements (TEs) may play an outsized role in establishing such incompatibilities because of their ability to rapidly proliferate. Studies of hybridization across diverse systems have revealed complex patterns of increased TE expression and transposition in hybrids [4–12]. However, it is not clear if increased TE activity is a general response to hybridization. To understand the effects of contrasting parental TE profiles during hybridization, intraspecific syndromes of hybrid dysgenesis can provide special insight. Hybrid dysgenesis is a phenomenon of sterility that arises during intraspecific hybridization when TE families are absent in one strain but abundant in another [13–16]. In particular, hybrid dysgenesis in *Drosophila* is induced when males carrying certain TE families fertilize females that lack them. Since the PIWI-interacting RNA (piRNA) system of genome defense in *Drosophila* relies on maternal deposition of piRNA to maintain TE repression across generations, if females lack a given TE family, they will also be incapable of transmitting corresponding piRNAs to their offspring [17, 18]. The combination of unrecognized TEs introduced to a naive genome via sperm and the absence of corresponding piRNAs in the egg results in TE activation and hybrid dysgenesis [18]. One well understood syndrome of hybrid dysgenesis is the *P-M* system of *D. melanogaster*. When P strain males carrying multiple copies of the *P* element DNA transposon are mated with M strain females that lack *P* elements, *P* elements transpose in the germline, cause DNA breaks and gonadal atrophy [13, 19–21]. These events primarily happen during early development of the offspring [22, 23], but can also happen at any stage [24]. In contrast, the *I-R* system of hybrid dysgenesis in *D. melanogaster* is associated with perturbation of meiosis in females and failure for eggs to hatch [25].

*D. virilis* is a species within the *Drosophila* subgroup and approximately 50 million years diverged from *D. melanogaster* [26]. Like other species within the *Drosophila* subgroup, *D. virilis* has six acrocentric chromosomes homologous to the six chromosome arms in *D. melanogaster* with a large expansion in satellite DNA making up approximately 40% of its genome [27]. This amount of satellite DNA is among the highest across the genus [27]. A unique syndrome of hybrid dysgenesis in *D. virilis* is observed in intraspecific crosses between males of an inducing strain (designated strain 160) and reactive strain females (designated strain 9) [16]. Similar to other systems, dysgenesis occurs when females lacking a given TE family are mated with males that carry them. Developmentally, it is more similar to the *P-M* rather than *I-R* system of dysgenesis in *D. melanogaster* because the events that cause sterility happen in the early germline [28, 29]. However, in contrast to the *P-M* system, sterility appears to be due to the mass activation of multiple TE families abundant in strain 160 but not strain 9. At least four elements are proposed to contribute to dysgenesis. *Penelope* and *Helena* are retrotransposons and *Paris* and *Polyphemus* are DNA transposons [30–34]. Three of these TEs (*Penelope*, *Helena* and *Paris*) have been previously shown to transpose and cause mutation in the germline of dysgenic progeny. The transposition of *Polyphemus* in the dysgenic germline has been proposed but not tested. In addition, activation of TEs during hybrid dysgenesis is associated with transposition of diverse TEs that are equally abundant between the two strains [30]. Whether co-mobilization of other elements occurs in the *P-M* system is a matter of dispute [35–37]. While the reactive strain carries mostly degraded copies, for likely active copies with very low divergence, inducer strain 160 carries 33, 56, 13 and 26 copies of *Polyphemus*, *Penelope*, *Helena* and *Paris*, respectively [38] (Additional file 2).

Besides mutation caused by transposition, it is an open question whether there are other genomic consequences of hybridization between strains that differ in TE content. In *Drosophila*, dysgenesis is associated with recombination in the male germline even though male meiosis normally occurs in the absence of crossing over. Male recombination events are known to form in clusters among sibling cohorts [39–41]. In the *P-M* system, induced male recombination is usually attributed to an increased rate of mitotic exchange induced by DNA damage [42]. These male recombination events often occur near *P* element insertions, require transposase, and are likely the byproduct of *P* element excision events that are repaired from the homolog [19, 20, 43–45]. In the female germline, meiotic recombination is shaped by the DNA damage response [46, 47], and thus ongoing transposition during meiosis might alter meiotic recombination by affecting the choice among various repair pathways for programmed double-strand breaks. Alternatively, if the timing of transposition is limited to early stages of germline development, the effect may be modest. Changes in female recombination rates were not initially reported during *P-M* hybrid dysgenesis [39, 48] but later studies found a slight increase [49–51]. However, others have identified no increase in female recombination rates caused by *P-M* hybrid dysgenesis but, rather, a redistribution towards regions with low recombination [52, 53]. Slightly increased rates of female recombination have also been reported for the *I*-*R* element system [48]. Recombination in the male germline is also a phenotype of hybrid dysgenesis in *D. virilis* where crossing over is typically absent [16, 54]. There have been no investigations of how hybrid dysgenesis influences female recombination—either mitotic or meiotic—in *D. virilis*. Therefore, we sought to determine how recombination in the female germline might be influenced by a syndrome of hybrid dysgenesis that appears to be driven by multiple TEs.

To determine how hybrid dysgenesis influences recombination genome-wide in *Drosophila* females, fine-scale genetic maps are required. *D. virilis* genetic maps have been obtained only with a limited number of markers which show that the rate of recombination in *D. virilis* is significantly higher than in *D. melanogaster* even though previously estimated rates also differ between studies [55–57] (Table 1). Here, we provide the first fine-scale genetic map for *D. virilis* using thousands of genotypic markers. Using this map, we investigate differences in both crossover (CO) frequency and distribution in the hybrid dysgenesis syndrome of *D. virilis*. As has been observed in male syndromes of hybrid dysgenesis, we identify multiple cases of clustered recombination events that occur within sibling cohorts, indicating mitotic recombination. Using a novel estimation approach, we show the probability of inheriting mitotic crossover chromosomes is elevated in the progeny of dysgenic females. Furthermore, these clustered recombination events can induce loss-of-heterozygosity and are associated with *Paris* and *Polyphemus* DNA transposons. In the case of *Polyphemus,* we also demonstrate that excision occurs during dysgenesis and provide the first direct evidence for transposition-induced DNA damage as the cause of hybrid dysgenesis in this syndrome. Interestingly, we find no significant differences in meiotic recombination between genetically identical dysgenic and non-dysgenic female progeny. Thus, meiotic recombination appears robust to TE activation during hybrid dysgenesis in *D. virilis*. This suggests that the effects of DNA damage during early development are not sufficient to trigger changes in the control of recombination later during meiosis.

**Table 1:** Genetic map lengths of *D. virilis* chromosomes in centiMorgans reported in previous studies and this study

	Chromosome					
	X	2	3	4	5	6
Gubenko & Evgen’ev (1984)	170	257	145	108	203	1
Huttunen et al. (2004)	–	118	125	147	60	–
Current study	143.5	160.9	139.6	148.3	140.0	–

## Results

### Crossover detection by sequencing

To identify recombination events in reciprocal F1 dysgenic and non-dysgenic progeny, F1 females were backcrossed to reactive strain 9. By sequencing backcross (BC1) progeny, we identified the recombination events that occurred under the dysgenic and non-dysgenic condition in the germline of F1 females. F1 dysgenic and non-dysgenic female progeny have identical nuclear genotypes, which enables a controlled comparison of the effects of TE mobilization on the recombination landscape. There is also a high amount of variation in the severity of dysgenesis within females. Many F1 dysgenic females are sterile while others have reduced fertility due to gonadal atrophy. However, there is a subset of F1 dysgenic progeny that do not have gonadal atrophy or decreased fertility associated with the dysgenesis phenotype. This provides a natural three-way comparison of recombination rates in F1 progeny with no TE activation (non-dysgenic), TE activation and severe germline damage (low fecundity dysgenic), and TE activation with germline recovery (high fecundity dysgenic). In total, 828 BC1 female progeny were sequenced at sufficient depth (0.15X average coverage) to map recombination breakpoints; 132 samples had fewer than 10,000 reads (< 0.005X coverage) and were not included in our analysis. 275 BC1 progeny were sequenced from 20 F1 non-dysgenic females, 311 BC1 progeny were sequenced from 66 low fecundity F1 dysgenic females, and 242 BC1 progeny were sequenced from seven high fecundity F1 dysgenic females. Across all samples, the multiplexed shotgun genotyping (MSG) pipeline identified a total of 1,150,592 quality-filtered SNPs between the two parental genomes with an average distance of 136 bp between SNPs. The MSG Hidden Markov Model (HMM) uses relative mapping abundance of reads that are uniquely derived from one of the two parental strains [58]. Using this information, combined with the crossing scheme, it provides genotype probabilities for each SNP. For each sample, and at each SNP, the HMM provided a genotype probability of the BC1 progeny sample being either homozygous for strain 9 (the strain that the F1 progeny were backcrossed to) or heterozygous. CO breakpoint intervals were then defined based on local genotype probability calls that switch from greater than 95% to less than 5% along the chromosome. The median CO breakpoint interval length calculated by the MSG HMM was approximately 18 kb and 84% of all COs localized within a span of 50 kb or less. Seventeen CO breakpoint intervals were approximately ~ 1 Mb but those were in samples with low read counts near the 10,000 read cutoff for samples allowed in the analysis.

### A high-resolution genetic map of *D. virilis*

Previous studies indicate that the genetic map of *D. virilis* is approximately three times larger than the genetic map of *D. melanogaster* [56, 57]. Critically, the map lengths obtained in those two studies are quite different (Table 1), perhaps due to the limited number of genetic markers used in previous studies. Our combined sample has a sufficient density of markers to provide the first high-resolution recombination map for *D. virilis*. Combining results from all samples to construct our map was reasonable, since the effects of dysgenesis were non-significant (see below).

The total genetic map length of *D. virilis* estimated in our combined sample is 732 cM (centiMorgans) or 2.5 times longer than the genetic map length of *D. melanogaster* [59] (Table 1). The genetic map length estimated in the current study is more than 100 cM shorter than the first detailed genetic map of *D. virilis* [56] (Table 1). This may be partly explained by our stringent exclusion of problematic genomic regions. However, comparing chromosomes that were characterized in all three studies (2, 3, 4, and 5), our estimate for cM is within or very close to the upper estimate of the two prior studies. In addition, our cM estimates were more uniform across the chromosomes, which are all fairly similar in physical length. As expected, the genetic map length of each chromosome in our study correlates with physical length (*R*^*2*^ = 0.851, *p* = 0.025). There is no significant correlation in the two prior studies (*R*^*2*^ = 0.079, *p* = 0.72 [57]; *R*^*2*^ = 0.358, *p* = 0.28 [56], excluding the 6th chromosome). The differences in recombination rates between *D. virilis* and *D. melanogaster* might be explained by their differences in genome size. The estimated genome size of *D. virilis* is roughly twice the size of the *D. melanogaster* genome (404 vs 201 Mb) [27]. Thus, across the entire genome, the average rate of recombination in *D. virilis* is 1.8 cM/Mb and similar to the average recombination rate of 1.4 cM/Mb in *D. melanogaster*. However, close to half of the *D. virilis* genome is comprised of satellite DNA, with large blocks of pericentromeric heterochromatin where little or no crossing over takes place [27, 60]. Thus, the *D. virilis* euchromatic portion of the genome, where most COs take place, is approximately 206 Mb in length and the length of the reference genome with usable markers for this study was 155 Mb. Accounting for just euchromatic regions in both species, the average rate of recombination in euchromatin in *D. virilis* is more than twice as high as *D. melanogaster* based on the length of span of usable markers for this study (4.6 cM/Mb vs. 1.8 cM/Mb). One possible reason for a higher rate of recombination in *D. virilis* may be the fact that pericentric heterochromatin comprised of satellite DNA may shield the chromosomes arms from the suppressive centromere effect [61]. The expansion of pericentric satellite DNA in *D. virilis* may reduce the spread of the centromere effect into the euchromatic regions; the *D. melanogaster* genome has less expansive satellite DNA arrays and the centromere suppression of recombination reaches far into the euchromatic regions of this species [59, 62] Nonetheless, we do see some suppression of recombination proximal to the centromeres within the span of informative genotypic markers suggesting the centromere effect is present in *D. virilis* and these satellites are not sufficient to fully suppress the centromere effect in proximal euchromatin (seen below in the distribution of CO events).

Recombination rates are often correlated with TE density and other sequence features, such as GC content, simple non-satellite motifs, and nucleotide polymorphism [59, 63, 64]. In *D. virilis*, TE density shows a strong negative correlation with recombination rate but this becomes weaker when non-recombining regions are removed (Table 2, Additional file 1: Figure S1). The similar pattern of weak correlation between TE density and recombination when regions without recombination are removed is also seen in *D. melanogaster* [65], where TEs mostly accumulate in non-recombining pericentromeric heterochromatin [66, 67]. Recombination rates in *D. virilis* also appear to be weakly correlated with GC content and gene density as not all chromosomes show significant correlations with either sequence parameter (Table 2). This may be due to unusual patterns of recombination along the length of the chromosome (discussed later). Simple repeats and SNP density between the two strains show significant positive correlations with recombination rate on all chromosomes even after removal of non-recombining regions. Nucleotide diversity is frequently correlated with recombination rates [63, 64] and the strong correlation between SNP density and recombination in our data is consistent with this pattern (Additional file 1: Figure S1) [66, 67].

**Table 2:** Pearson’s correlation coefficients (*R*) and *p*-values between rates of recombination and sequence parameters calculated in 250 kb intervals

Sequence Parameter		Chromosome					Total	Total minus Zero CO
		X	2	3	4	5		
TE Density	R	−0.47	−0.47	−0.33	− 0.44	−0.49	− 0.44	−0.14
	*p*	*<0.001*	< 0.001	< 0.001	< 0.001	< 0.001	< 0.001	<1E-10
Gene density	R	0.31	0.21	0.12	0.32	0.33	0.19	0.03
	*p*	< 0.001	0.012	0.222	< 0.001	< 0.001	< 0.001	0.506
Simple motifs	R	0.44	0.43	0.31	0.32	0.54	0.39	0.177
	*p*	< 0.001	< 0.001	< 0.001	< 0.001	< 0.001	<1E-10	< 0.001
SNP Density	R	0.64	0.553	0.60	0.67	0.65	0.62	0.49
	*p*	<1E-10	<1E-10	<1E-10	<1E-10	<1E-10	<1E-10	<1E-10
GC Content	R	0.08	0.35	0.11	0.18	0.33	0.23	0.15
	*p*	0.372	< 0.001	0.263	0.079	< 0.001	< 0.001	< 0.001

### No modulation of recombination rates during hybrid dysgenesis

**Fig. 1:**
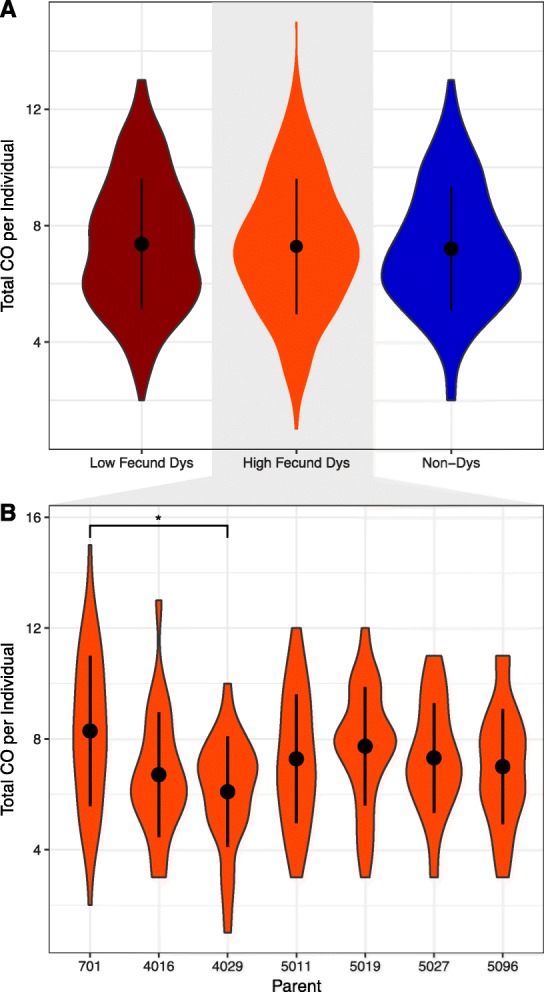
The distribution of the total crossover (CO) count observed in individual BC1 progeny with the mean and standard deviation. The mean for each group is represented with a black dot and the standard deviation is the black line. **a** The distribution of the total CO count per BC1 progeny of low fecundity dysgenic, high fecundity and non-dysgenic F1 mothers. **b** The distribution of CO count per BC1 progeny of each high fecundity dysgenic mother with mean and standard deviation. Asterisks denotes statistical significance by least square means (*p* < 0.05). Progeny from mother 701 had a higher average CO count than progeny from other mothers while progeny from mother 4029 exhibited a lower average CO count

To compare recombination rates in dysgenic and non-dysgenic females, we constructed a full mixed-effects likelihood model using the lme4 R package [68, 69]. The direction of the cross (dysgenic vs. non-dysgenic) and F1 collection batch (pilot vs. second experiment) were treated as fixed effects; the F1 female of origin was treated as a random effect. In the full model, we find no difference in the total number of COs between the pilot and second experiment (χ^2^_1_ = 0.10, *p* = 0.755). This suggests that there were no effects of library construction procedure and justifies combining data sets. There is significant variation in fecundity among dysgenic females. Some females are completely sterile, some have significantly reduced fecundity and others have essentially normal levels of fecundity. Since females with significantly reduced fecundity might have experienced greater levels of TE mobilization, we designated females as either low fecundity or high fecundity (see methods). No effect was found for fecundity in dysgenic flies on CO numbers (χ^2^_1_ = 2.02, *p* = 0.155). Importantly, dysgenesis did not have a significant effect on total CO numbers (χ^2^_1_ = 0.45, *p* = 0.506) with nearly identical means in CO number between dysgenic and non-dysgenic progeny (Fig. 1a). There was a marginal interaction between dysgenesis and batch (χ^2^_1_ = 3.17, *p* = 0.075), but this appears driven by a single high fecundity dysgenic F1. This F1 female, designated 701, revealed a larger mean CO number in comparison to the other high fecundity dysgenic females (8.3 COs, least squares mean contrast, *p* = 0.021, Fig. 1b). Without the 701 female, the interaction between dysgenesis and batch is non-existent (χ^2^_1_ = 0.06, *p* = 0.803) and dysgenesis continues to have no effect on CO numbers (χ^2^_1_ = 0.03, *p* = 0.874). Overall, the full model revealed that the parent of origin had minimal effect on CO number (variance < 0.0001).

**Fig. 2:**
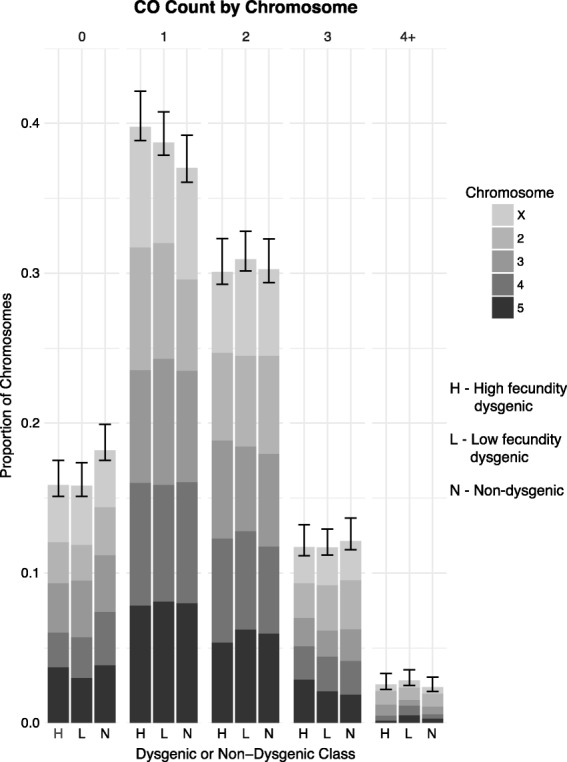
The proportion of chromosomes grouped by CO count in BC1 progeny of high fecund dysgenic, low fecund dysgenic, and non-dysgenic F1 mothers. 95% confidence intervals were calculated by sampling BC1 progeny by bootstrapping 1000 times

The higher recombination rate per Mb in *D. virilis* in comparison to *D. melanogaster* is due to a higher number of COs on each chromosome. In *D. melanogaster*, chromosome arms typically have zero, one, or two COs with three COs on a single chromosome arm being rare [70]. In contrast, a chromosome with three or more COs is common in *D. virilis*, in both dysgenic and non-dysgenic directions of the cross. Chromosomes with five COs were also observed (Fig. 2). CO counts per chromosome were highly similar between the progeny of dysgenic and non-dysgenic F1 females (χ^2^_4_ = 0.529, *p* = 0.97). Likewise, there was also no difference between dysgenic mothers if they were high fecundity (χ^2^_4_ = 3.70, *p* = 0.45) or low fecundity (χ^2^_4_ = 3.45, *p* = 0.49).

**Fig. 3:**
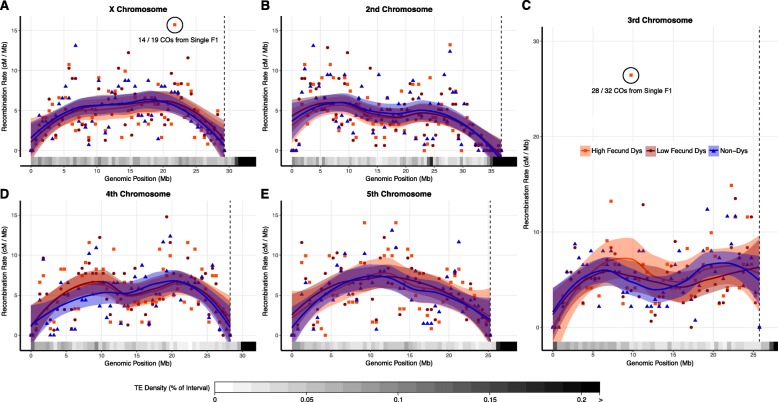
Loess smoothed splines of the recombination rate along the length of each chromosome in *D. virilis* from the telomere (left) to the centromere (right) with standard error and corresponding TE density. The dotted line represents the centromere effect of recombination suppression as recombination = 0 from the line to the end of the sequence. The rate of recombination and TE density were calculated in 500 kb intervals in F2 progeny of low fecund dysgenic, high fecund and non-dysgenic F1 mothers for the **a** X chromosome, **b** 2nd chromosome, **c** 3rd chromosome, **d** 4th chromosome, and **e** 5th chromosome. Two identified clusters of recombination are highlighted in the circled regions

We also examined the distribution of recombination events along the length of each chromosome between non-dysgenic flies, high fecundity dysgenic flies, and low fecundity dysgenic flies. There were no major changes in the distribution of recombination along the length of the chromosomes (Fig. 3). The chromosomal recombination rates between all three groups are strongly correlated (Additional file 1: Table S1). Interference plays a role in determining CO positioning. Therefore, we determined whether interference was altered by dysgenesis by comparing the distribution of distances between crossovers identified in the progeny of dysgenic and non-dysgenic flies. We found no differences in the distribution of crossovers for any chromosome (Mann-Whitney U test, *p* > 0.5). Overall, we find no differences in the recombination landscape between dysgenic and non-dysgenic F1 mothers in *D. virilis* at the global level. This suggests there is little to no feedback between putative activation of the DNA damage response during dysgenesis in *D. virilis* and the modulation of meiotic recombination.

### A signature of early DNA damage and mitotic crossing over in dysgenesis

**Fig. 4:**
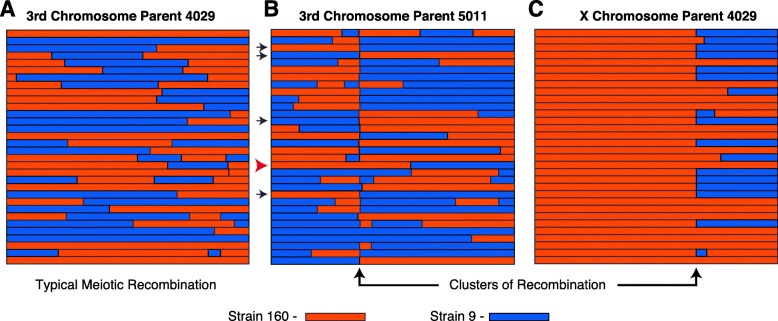
Haplotypes of BC1 progeny from a single high fecundity dysgenic mother. **a** Haplotypes of the third chromosome in progeny of the 4029 F1 mother is typical of most chromosomes with no cluster of recombination. **b** Haplotypes of the third chromosome in progeny of the 5011 F1 mother identify a common recombination breakpoint in most of the progeny and reciprocal products of recombination in equal frequency (Binomial test, *p* > 0.05). Arrows indicate samples that were tested for retention of the *Polyphemus* insertion. Black arrows indicate absence of the *Polyphemus* insertion. The red arrow indicates a non-recombinant sample with an excision scar identified by sequencing. **c** Haplotypes of the X chromosome in progeny of the 4029 F1 mother indicate a common recombination breakpoint in half of the progeny and extreme transmission distortion of the distal portion of the chromosome (227 markers 0.5–21.4 MB, Binomial test, *p* < 1E-07). The proximal region of the chromosome shows no transmission distortion (86 markers 21.5–29.0 Mb Binomial test, *p* > 0.5)

Despite observing no significant effect of dysgenesis on meiotic recombination rates, we observed several genomic regions that exhibited a much higher apparent number of COs during hybrid dysgenesis. For example, within a 500 kb region on the third chromosome, the apparent recombination rate was 26 cM/Mb, nearly twice as high as any other interval within the genome (Fig. 3c, circled region). 32 COs in this region were identified as arising from dysgenic F1 females compared to a single CO identified from non-dysgenic mothers. The COs in this interval provided evidence for a mitotic recombination cluster because the majority (28/32) were identified in the progeny of a single highly-fecund dysgenic F1 mother designated 5011. The mitotic recombination event is easily visible in the genotypes of the BC1 progeny of F1 mother 5011 in comparison to the BC1 progeny of another female with no cluster of recombination on the same chromosome (Fig. 4a-b). Reciprocal CO products were observed with equal frequency among the BC1 progeny of F1 mother 5011 (χ^2^_1_ = 0.13, *p* = 0.727, Fig. 4b) indicating no transmission bias among recombinant gametes. Additional unique COs were detected along the entire length of the third chromosome proximal and distal from the recombination cluster. The high frequency of recombinants at the same location identified among this cohort of BC1 progeny likely indicates an event in the early germline of the F1 female 5011.

Another recombination cluster was identified on the X chromosome, approximately 21.7 Mb from the telomere. In this region, there was an effective recombination rate of 15.7 cM / Mb (Fig. 3a, circled region). Once again, the vast majority of COs within this 500 kb interval are part of another cluster of recombination attributed to a single highly-fecund dysgenic F1 female designated 4029. The cluster of recombination is revealed in only half of the progeny of the F1 mother 4029 (Fig. 4c). Interestingly, no additional COs were detected in the portion of the X chromosome distal from the recombination event and all markers in the distal portion were heterozygous. The extreme excess of heterozygosity on the X chromosome in the BC1 progeny indicates transmission distortion of the strain 160 genotype distal from the CO from the 4029 mother (χ^2^_1_ = 32, *p* = 0.141E-08, Fig. 4c). Markers proximal to the cluster of recombination show no transmission distortion (χ^2^_1_ = 0.13, *p* = 0.727, Fig. 4c). Moreover, crossing over was found in the proximal portion of the X. Thus, we conclude there was germline loss of heterozygosity (rather than meiotic drive) for the distal region of the chromosome.

These two clusters of recombination were identified based on their observed higher rates of recombination in the dysgenic germline. We infer the clusters are mitotic recombination hotspots because the chromosomes with the focal recombination event were exclusively found to be derived from a single F1 mother. Additional mitotic COs may be indistinguishable from meiotic recombination since such events may be rare and are only evident if the events occur early enough in development and are associated with depletion of other non-CO germline stem cells. To uncover additional evidence for other mitotic COs in our data, we screened for clusters of recombination among cohorts by identifying CO events within the same 100 kb interval in four or more progeny of a single F1 mother. Using these criteria, we identified five additional candidate mitotic recombination clusters in progeny from dysgenic mothers and one additional candidate in progeny from a non-dysgenic mother (Table 3). Four of these six additional putative clusters of recombination were also associated with transmission distortion of a single genotype in a significant portion of the chromosome and no additional COs detected in the distorted region (Table 3, Additional file 1: Figure S2).

**Table 3:** Clusters of recombination identified in the BC1 progeny

F1 Female Identifier	Chromosome	Position (Mb from Telomere)	CO Progeny / Total Progeny	Distortion Genotype	TEs detected in Strain 160
3^a^	X	17.7	4 / 29	None	*Polyphemus*
111	3	25.1	4 / 11	Strain 9^b^	*Polyphemus*
4013	X	11.4	4 / 13	Strain 160	None
4029	X	21.3	14 / 19	Strain 160	None
5011	3	9.8	28 / 32	None	*Polyphemus*
5019	3	20.2	10 / 30	Strain 9^b^	None
5022	3	23.6	5 / 11	Strain 9^b^	*Paris*
5089	X	23.4	4 / 11	Strain 160	*Polyphemus* ×2, Paris

^a^The F1 female is non-dysgenic

^b^Transmission distortion occurred in some of the progeny. The rest appear normal and probably did not inherit a mitotic CO chromatid

To address whether activation of TEs during dysgenesis played a role in causing clustered mitotic recombination events, we generated a de novo PacBio assembly for strain 160 and determined whether regions of the 160 inducer chromosomes where recombination clusters were identified contained intact copies of TEs implicated in hybrid dysgenesis (*Penelope*, *Polyphemus*, *Helena*, and *Paris*) (Additional file 2). Active versions of these TEs are absent in strain 9 and their activation during hybrid dysgenesis may induce DNA damage on the 160 chromosome for subsequent repair via mitotic recombination. Of these, *Paris* and *Polyphemus* are the most likely associated with chromosome breaks since they are DNA transposons capable of excision. By examining the PacBio assembly of strain 160, we found that five clusters of recombination contained an intact insertion for a TE known to be absent in strain 9 and present in strain 160 within the defined boundaries of the CO (Table 3). Three clusters were associated with *Polyphemus* elements in strain 160. One cluster was associated with a *Paris* element and a fifth cluster on chromosome X contained both elements (Table 3). We find these clusters of recombination are enriched in DNA transposons (*Paris* and *Polyphemus*) implicated in hybrid dysgenesis relative to the rest of the genome (Binomial test, *p* < 1E-07). To determine whether mitotic recombination events are directly associated with excision during dysgenesis, we performed PCR and sequencing on original DNA samples used for Illumina genotyping of the BC1 progeny of F1 mother 5011 with primers that flanked the *Polyphemus* insertion on chromosome 3. Examination of the one individual that retained the strain 160 haplotype across the CO breakpoint indicated that even though there was no recombination event for that sample, excision of *Polyphemus* was identified in the lesion left by the target site duplication. We also tested for presence of *Polyphemus* in four recombinant samples and confirmed that *Polyphemus* was absent in all four samples. Crossover events initiated from an excision are expected to be repaired off the non-insertion chromosome and, thus we were not able to find direct evidence of an excision scar in the four recombinants lacking the *Polyphemus* element. Nonetheless, these results support the conclusion that this particular *Polyphemus* insertion was activated in female 5011 and this was associated with a cluster of mitotic recombination. Overall, our results suggest clusters of recombination occur more frequently in dysgenic relative to non-dysgenic females and often occur in regions containing intact copies of two DNA transposons (*Polyphemus* and *Paris*) associated with hybrid dysgenesis. We note that eight mitotic events on only two of five chromosome arms represents significant enrichment on the X and 3rd chromosome (Binomial test, *p* < 0.01). We attribute this to heterogeneity in element activity, as has been found for *P* elements in the *P-M* system of hybrid dysgenesis [71].

Since we identified multiple mitotic clusters of recombination in dysgenic crosses, we sought to more formally determine whether there was evidence for a statistically significant higher rate of mitotic recombination formation in dysgenic females. Since the criteria for identifying a cluster was based on observing four or more individuals with a CO within a given span, it was necessary to account for variation in cohort size. We achieved this by developing a likelihood model where the probability of observing a set of chromosomes providing evidence of a cluster within a cohort was a function of the probability that a mitotic event occurs on that chromosome within an F1 female (α) and the probability of observing that event on a given chromosome (β) among the sequenced progeny (penetration of the event among the cohort). We considered two, three and four parameter models where either α or β would be the same under dysgenesis or non-dysgenesis, or there would be a unique value depending on condition. Using nested likelihood ratio tests, we find support for a three-parameter model with separate β estimates for dysgenic and non-dysgenic mothers and a shared α estimate over the two-parameter model (α = 0.12, β_Dys_ = 0.78, β_NonDys_ = 0.11, LRT, χ^2^_1_ = 51.6, *p* = 6.92E-13, Additional file 1: Table S2). Though more mitotic clusters were observed derived from dysgenic mothers, and the three-parameter model where α is estimated separately estimates a more than four-fold increase of mitotic cluster formation during dysgenesis (α_Dys_ = 0.12, α_NonDys_ = 0.026, Additional file 1: Table S2), support for this model was not significant relative to the two-parameter model (LRT, χ^2^_1_ = 3.39, *p* = 0.066, Additional file 1: Table S2). Finally, we do not find support for a four-parameter model over the three-parameter model with separate β estimates (LRT, χ^2^_1_ = 1.88, *p* = 0.170, Additional file 1: Table S2). Overall, these results support a similar baseline rate of occurrence of mitotic events in the dysgenic and non-dysgenic germlines. However, the frequency of chromosomes that are transmitted with mitotic damage is greater in the dysgenic germline. Thus, we conclude that the total transmission rate of mitotic damage (α * β) is more than six times greater in the dysgenic germline (0.096 mitotic COs per gamete in dysgenic, 0.014 mitotic COs per gamete in non-dysgenic).

## Discussion

In the hybrid dysgenesis syndrome *D. virilis*, diverse TEs are known to transpose when active families that are paternally inherited are absent in the maternal genome [30]. Here we evaluate the effect of this genomic clash on the recombination landscape.

To evaluate the consequences of parental TE asymmetry on recombination, we obtained the first high-resolution genetic map of *D. virilis*. Combined with recombination studies in *D. simulans*, *D. mauritiana*, *D. yakuba*, *D. persimilis*, *D. miranda*, *D. serrata*, *D. mojavensis*, and others [62, 72–79], our genetic map of *D. virilis* will aid future studies of the evolution of recombination in *Drosophila*. Of note is the high rate of recombination in *D. virilis* in comparison to other species, especially *D. melanogaster*. Recombination rates in species of *Drosophila* frequently peak in the middle of the chromosome arm and decrease towards the centromere and telomere [62]. However, the distribution of recombination on the second, third, and fourth chromosomes in *D. virilis* resembles a bimodal distribution (Fig. 3). The bimodal distribution may be the result of the exceptionally high recombination rates in *D. virilis*. When two or more crossovers on a single chromosome is common, interference preventing CO formation in close proximity would spread COs more evenly along the length of the chromosome.

This study is one of the few to examine the effects of asymmetric TE inheritance on the meiotic recombination landscape, and the first to do so using the hybrid dysgenesis syndrome in *D. virilis*. Results from previous studies of hybrid dysgenesis in *D. melanogaster* were conflicting as some found no effect on female recombination [39, 48] while others found increases in the recombination rate [49–51] or changes in the distribution of recombination [52, 53]. In addition to reporting findings using the dysgenic syndrome of a different species, ours is also the first study to investigate the effects of hybrid dysgenesis on recombination using high-throughput genotyping rather than phenotypic markers. This allows a greater insight into the fine-scale changes in recombination rates and distribution that may previously have escaped unnoticed.

**Fig. 5:**
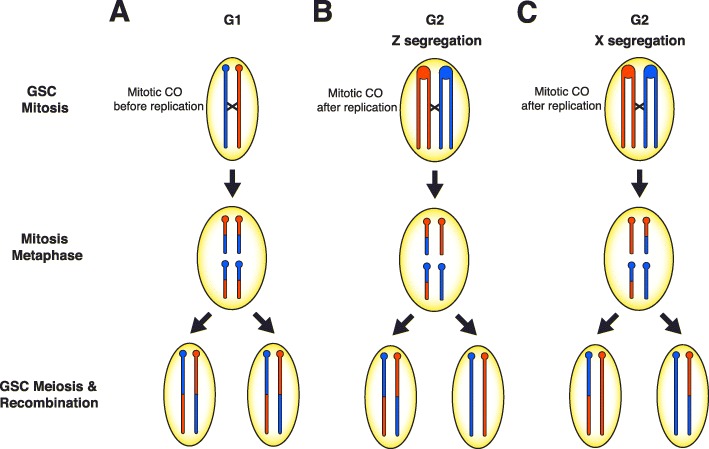
Models to explain the clusters of recombination on the third and X chromosomes in the progeny of two high fecundity dysgenic mothers. In the 5011 F1 female, a mitotic crossover (CO) either occurred **a** prior to DNA replication in the early developing germline resulting in two daughter cells with the CO or **b** after DNA replication, followed by a pattern of Z segregation so that one daughter cell has both CO chromatids. Oocytes produced by these germline stem cells will transmit the CO as reciprocal products as seen if the CO occurred in G1. **c** A mitotic CO in the 4029 F1 female occurred after DNA replication in the developing germline and each daughter cell received one CO chromatid and one non-CO chromatid according to a pattern of X segregation. This results in a loss of heterozygosity (LOH) in the distal portion of the chromosome to resemble transmission distortion and recombination events are not detectable

In contrast to finding no effect on meiotic recombination, we identified an elevated rate of observing clusters of recombination in the progeny of dysgenic females. We interpret these clusters to originate from mitotic CO events that occur early in germline stem cell (GSC) development (Fig. 5). This interpretation is supported by the fact that during hybrid dysgenesis, the damaging effects of transposons are often observed in the germline during early development [22, 23, 28]. We find germline cell death and TE activation during early development does not have any significant lingering effect on meiotic recombination in the adult female germline. Meiotic recombination is also not affected by asymmetric TE expression from hybrid dysgenesis which persists into the adult germline in *D. virilis* [38]. However, double-stranded breaks (DSBs) produced as an outcome of transposition may be repaired by one of several mechanisms, including homologous recombination via mitotic crossing over prior to meiosis. Mitotic crossing over can explain several different observations in our data. In the case of the cluster of COs on the third chromosome in F1 mother 5011, excision of a *Polyphemus* DNA transposon may have produced a DSB repaired via homologous recombination in the mitotic germline, which occurred in G1 prior to DNA replication within a developing GSC (Fig. 5a). In this scenario, reciprocal products of the CO would appear in all daughter cells descended from this GSC and reciprocal products would be observed, on average, in equal frequency among gametes. Alternatively, a mitotic CO may have occurred after DNA replication in G2 prior to mitosis in the germline of the 5011 mother (Fig. 5b). During mitosis, the chromatids segregated according to Z segregation [80, 81] such that reciprocal CO products were transmitted to one daughter cell while the other daughter cell would have received the non-CO chromatids. Non-CO GSCs must have been retained within the 5011 mother because there are several progeny without the common CO product. However, the limited number of progeny lacking either of the reciprocal CO chromatids indicates a depletion of other non-CO GSCs. In either case, we attribute the high frequency of recombinant chromatids arising from mitotic crossing over to an early crisis in GSC survival due to hybrid dysgenesis, followed by re-population of the GSCs mainly from descendants of a single cell carrying the CO chromatids. GSCs marked with the mitotic CO were thus able to recover and rescue fertility after hybrid dysgenesis in the high fecundity female. This is consistent with the observation that hybrid dysgenesis is associated with an early phase of germline depletion [29].

Likewise, mitotic CO can explain a different recombination cluster on the X chromosome, with different consequences, that likely occurred in the early developing germline of the 4029 mother (Fig. 5c). In this case, there was apparent loss-of-heterozygosity (LOH) distal from the CO. This mitotic CO event likely would have occurred after DNA replication in G2, with a pattern that has been designated X segregation, in contrast to Z segregation [80, 81], resulting in each daughter cell receiving one chromatid with the CO and one without. X segregation induces homozygosity and LOH between the two inherited chromosomes, specifically in the regions distal from the CO. This LOH would be responsible for failure to detect additional meiotic COs derived from the homozygous distal region. The complete transmission distortion of the distal region suggests severe depletion of GSCs with the reciprocal mitotic CO products. Again, this is consistent with hybrid dysgenesis causing a severe reduction of GSCs, followed by re-population from even a single GSC and restoration of fertility in the high fecundity female. Interestingly, the bounds of the mitotic CO derived from the 4029 mother do not contain intact dysgenesis-associated TEs nor any other intact TEs in the strain 160 genome. This mitotic CO may therefore have been the product of a new TE insertion in the genome of the 4029 mother. LOH is also observed among several clusters of recombination and most of these clusters are associated with either *Polyphemus* or *Paris* DNA transposons (Table 3, Additional file 1: Figure S2). LOH via mitotic recombination is observed after DNA damage or chromosomal breakage in cancer cells [82] and in yeast recombination studies [83]. The greater number of mitotic recombination events with transmission distortion observed in our data is consistent with previous observations of non-random segregation of chromatids in clonal analysis; chromatids involved in mitotic exchange are more likely to segregate into separate daughter cells (X segregation) than the same daughter cell (Z segregation) in mosaic analyses [80, 81]. Likewise, transmission distortion is frequently observed during hybrid dysgenesis [40, 41]. Our study therefore links transmission distortion via mitotic recombination and LOH within female germlines as a result of hybrid dysgenesis.

The number of observed mitotic CO events identified in dysgenic progeny is interesting because the crossing over pathway is least likely to repair non-programmed DSBs [84]. Mitotic COs only account for ~ 1% of all COs detected in our dataset and contribute minimally to the genetic map length (Additional file 1: Figure S3). Interestingly, the mitotic exchange rate is similar to the rates of male recombination under *P* element hybrid dysgenesis [20, 41]. Other pathways of DNA damage repair including non-homologous end joining and single-strand annealing are probably more common but undetectable in our assay. Rates of mitotic crossing over may also be higher than estimated since many mitotic COs would not meet our criteria for identification because many dysgenic mothers produced fewer than four offspring. Finally, a limitation to our study is that we are only able to analyze the recombinational outcomes from surviving gametes. Moreover, because we achieved high-throughput with a shallow-sequencing protocol, we were unable to detect possible changes in non-CO gene conversion profiles or crossing over in heterochromatic regions. Future studies with long reads and deeper sequencing across samples may yet identify additional consequences that are resolved through alternative repair pathways.

## Conclusion

Modulation of recombination by hybrid dysgenesis may occur through different mechanisms. First, recombination could be directly initiated by DSBs that arise from either TE insertion or excision. Second, DSBs caused by TE activity could modulate global recombination rates through DNA damage signaling. Overall, despite evidence for DNA damage associated with transposon excision and ensuing mitotic recombination, we found no major differences in the distribution and frequency of meiotic recombination in *D. virilis* under hybrid dysgenesis. The DNA damage response has a critical role in regulating meiotic recombination [46, 47, 85]. DNA damage response regulators such as *p53* and *chk2* also influence the fate of GSCs during hybrid dysgenesis [86]. The incomplete penetrance of hybrid dysgenesis in *D. virilis* may arise from cell to cell variation in the total amount of DNA damage or in stochastic variation in the DNA damage response. However, we found no differences in recombination rates between dysgenic flies with minimal germline atrophy (high fecundity) and severe germline atrophy (low fecundity). This suggests that DNA damage signaling activated by dysgenesis does not modulate meiotic recombination. This is likely due to the fact that most TE activity happens in GSCs during early development [23, 28]. By the onset of meiosis, the harmful effects of TE activity during dysgenesis may have likely subsided. In *D. virilis*, TE suppression is restored by adulthood in dysgenic progeny via re-establishment of asymmetric piRNAs and the negative impacts of dysgenesis disappear in subsequent generations [38]. This suggests that TEs likely produce few DSBs during meiosis in the *D. virilis* hybrid dysgenesis model. We thus conclude that the timing of transposition is an important factor that determines how TEs influence recombination. In the future, it will be worth investigating whether recombination is also robust to transposition that occurs closer to the initiation of meiotic recombination.

## Methods

### Fly stocks and crosses

The hybrid dysgenesis syndrome in *D. virilis* is observed in crosses between reactive strain 9 females and inducer strain 160 males and the severity of dysgenesis is measured by the fecundity of the resulting progeny [16, 38]. The study was performed in two stages. A smaller pilot study was performed first, followed by a larger second study that incorporated additional optimization to increase throughput (see full description of sample counts in the supplementary data). We observed no significant differences between these two experiments, so we combined results for final analysis. For both experiments, each strain and all subsequent crosses were maintained on standard media at 25 °C. Prior to creating dysgenic and non-dysgenic hybrids, strain 160 and strain 9 were each inbred for 10 generations by single sib-pair matings. For each direction of the cross, approximately 20 4- to 6-day old virgin females of one strain and 20 2- to 10-day old males of the other strain were crossed *en masse* in bottles for 6 days. Strain 160 males crossed with strain 9 females induce dysgenesis in the F1 generation while the reciprocal cross yields non-dysgenic F1 progeny. Reciprocal crosses yield F1 flies with identical genetic backgrounds, with the exception of the mitochondrial genome. By comparing the recombination landscape between F1 females with identical nuclear genotypes, we obtain a robust analysis of how hybrid dysgenesis influences recombination that effectively controls for genetic background. Individual virgin F1 females, 4 days post-emergence, were backcrossed in single vials to two or three 2- to 10-day old strain 9 males and maintained in vials for 6 days. Non-dysgenic females were only allowed to lay for four to 5 days due to their high fertility to prevent vial crowding. Because fertility was low in dysgenic females, and to increase sample size of progeny within cohorts, a second brood was obtained from dysgenic F1 females by transferring to another vial after 10 days in the second, larger experiment. These females were allowed to lay for an additional 4 days. We found no difference in recombination between first and second broods (see below), so the results were combined across broods. Female backcross progeny (BC1) were collected once per day and immediately frozen at − 20 °C. Between 12 and 20 sisters from each non-dysgenic F1 female was collected as a sibship. All female progeny of the dysgenic F1 backcrosses were collected. To ensure balance in sequencing autosomes and sex-chromosomes, only BC1 females were sequenced. The male BC1 progeny were counted in the larger second study to test for the effect of fecundity on meiotic recombination rates (see below).

There is a high amount of variation in fecundity in dysgenic females. Some females are completely sterile, others have only reduced fecundity and some even have high numbers of progeny. One approach to analyzing the effects of dysgenesis on recombination would be to sample only single daughters from each F1 female. However, this approach would not allow for the discovery of recombination events arising as clusters within the germline. Therefore, we elected to sequence cohorts of BC1 sisters, balancing our sequencing across cohorts with different levels of fecundity. To determine if there might be an effect of fecundity on recombination, all male and female BC1 progeny across the two broods from the second larger experiment were counted to measure the fecundity of the F1 mother. In some cases, dysgenic F1 females escape the effects of dysgenesis completely and produce as many progeny as non-dysgenic females (> 20 offspring). For these dysgenic F1 females, designated high fecundity, approximately 40 BC1 female progeny were subjected to recombination analysis by sequencing. Progeny produced by the low fecundity F1 dysgenic females were collected with cohort sizes ranging from one to 20 sisters with most cohort sizes less than ten. By sampling larger cohorts from high fecundity F1 dysgenic females, we sought to identify clustered recombination events derived within the germline of single females. Power to detect these events among a small cohort of sisters is low. By examining recombination in both high fecundity and low fecundity dysgenic females, we also obtained an additional comparison in the analysis of recombination landscapes between two outcomes of TE activation: TE activation with strong deleterious effects on fertility versus TE activation with no observable negative effects on fertility. For a full description of sampling, see Additional file 1: Table S3.

### DNA extraction, library preparation, and Illumina sequencing

Sequencing libraries were prepared in two batches using different protocols, with the second batch protocol altered to increase throughput. We found no differences in recombination rates between batches (see below), indicating results are robust to protocol differences. The first batch included 192 samples and library preparations were performed following the protocol outlined in [58] with minor modifications. Single flies were placed into a U-bottom polypropylene 96-well plate with lysis buffer and 3.5 mm steel grinding balls (BioSpec) then homogenized with a MiniBeadBeater-96 at 2100 rpm for 45 s. DNA extractions on homogenized tissue were performed with the Agencourt DNAdvance Genomic DNA Isolation Kit (Beckman Coulter) following the Insect Tissue Protocol. DNA quantification was spot checked on some samples and estimated to average 1–2 ng/μl. For each sample, 10 μl of genomic DNA was digested with 3.3 U of MseI in 20 μls of reaction volume for four hours at 37 °C, followed by heat inactivation at 65 °C for 20 min. FC1 and FC2 adaptors [58] (Additional file 1: Tables S4-S5) were ligated to the digested DNA with 1 U of T4 DNA ligase (New England Biolabs) in 50 μl of reaction volume at 16 °C for 5 h and inactivated at 65 °C for 10 min. The samples were pooled and concentrated using isopropanol precipitation (1/10 vol NaOAc at pH 5.2, 1 vol of 100% isopropanol, and 1 μl glycogen). The library was resuspended in 125 μl of 1X Tris-EDTA (pH 8). Adapter dimers were removed with 1.5X vol AMPure XP Beads (Agencourt) and ligated products were resuspended in 32 μl of 1X Tris-EDTA (pH 8). 200–400 bp DNA fragments were selected using a BluePippin (Sage Science). Size-selected fragments were cleaned using 2X vol of AMPure XP beads and resuspended 20 μl of 1X elution buffer (10 μM Tris, pH 8). Libraries were quantified using a Qubit fluorometer before an 18-cycle PCR amplification on bar-coded fragments with Phusion high-fidelity PCR Kit (New England Biolabs). The PCR products were cleaned using 1X vol of AMPure XP Beads.

For the larger second batch (768 samples), sequencing libraries were constructed with an optimized rapid DNA extraction protocol and an in-house Tn5 transposase similar to the procedure outlined in [87]. DNA was extracted using the *Quick*-DNA 96 kit (Zymo) and 1–2 ng of DNA was tagmented with Tn5 transposase stored at a concentration of 1.6 mg/ml with pre-annealed oligonucleotides. Tagmentation was performed in 20 μl reaction volumes containing 2 μl of 5X TAPS-DMF buffer (50 mM TAPS-NaOH, 25 mM MgCl_2_ (pH 8.5), 50% v/v DMF) and 2 μl of 5x TAPS-PEG buffer (50 mM TAPS-NaOH, 25 mM MgCl_2_ (pH 8.5), 60% v/v PEG 8000). Samples were incubated at 55 °C for 7 min then rapidly lowered to a holding temperature of 10 °C. Reactions were inactivated with 5 μl of 0.2% SDS followed by an additional incubation at 55 °C for 7 min. PCR-based barcoding was performed on 2.5 μl of sample tagmentation volumes using the KAPA HiFi HotStart ReadyMix PCR Kit (Thermo Fisher Scientific), 1 μl of 4 μM Index 1 (i7) primers (Additional file 1: Table S6), and 1 μl of 4 μM Index 2 (i5) primers (Additional file 1: Table S7) in 9 μl of reaction volume. The PCR thermocycling conditions were: 3 min at 72 °C, 2 min 45 s at 98 °C, followed by 14 cycles of 98 °C for 15 s, 62 °C for 30 s, 72 °C for 1 min 30 s. PCR-amplified samples were pooled and the pooled samples were cleaned using 0.8 X vol AMPure XP Beads. We size-selected DNA fragments 250–400 bp from the pooled sample on a BluePippin and cleaned using 1X vol of AMPure XP Beads.

All libraries were sequenced at the University of Kansas Genomics Core on an Illumina HiSeq 2500 Sequencer with 100 bp single-end sequencing. The first 192 samples were sequenced on two lanes using the Rapid-Run Mode while the Tn5-produced libraries were sequenced on two lanes using the High-Output Mode (summary of the output is in Additional file 3).

### DNA extraction, library preparation, PacBio sequencing and assembly

PacBio sequencing was performed on strain 160 after 10 generations of single-sib mating followed by re-validation for induction of dysgenesis. Females collected for DNA extraction were allowed to eclose over 10 days, aged for two additional days, starved for 12 h to evacuate the gut, then immediately frozen in liquid nitrogen. 500 mg of whole flies were then used for DNA extraction with Blood Cell and Culture Midi Kit (Qiagen) [88]. The mortar was pre-chilled with liquid nitrogen prior to grinding and the resulting fine powder was directly transferred into Buffer G2. DNA extraction was performed across 5 columns, using a total of 47.5 mls G2, 190 μl RNAse A (100 mg/ml) and 1.25 ml of Protease from the Qiagen Kit. 50 mls were placed in a 50 °C shaker overnight. After overnight incubation, debris was spun down and poured onto column. The elution was performed according to manufacturer’s instructions and precipitated with 0.7 volumes of isopropanol, followed by spooling with a glass rod and resuspending in 100 μl EB Buffer. The final DNA concentration was estimated with a Qubit to be 843 ng/μl, yielding approximately 85 μg. PacBio sequencing was performed by the University of Michigan DNA Sequencing Core.

Purified strain 160 DNA was used to generate PacBio SMRTbell libraries using the protocol: Procedure & Checklist 20 kb Template Preparation with BluePippin Size Selection. Briefly, approximately 10 μg of template was sheared using Covaris g-TUBES to obtain a 20–25 Kb target length. Sheared DNA was purified using pre-washed AMPureXP beads, analyzed for size and concentration, then treated with Exo VII to remove single stranded DNA, followed by DNA damage and end repair. End repaired DNA was then blunt ligated to adaptors to form SMRTbells and treated with Exo VII plus Exo III to remove any fragments that lack adaptors on both ends. SMRTbells were purified using pre-washed AMPureXP beads, analyzed for size and concentration, then run through a Sage Scientific Blue Pippin instrument with 0.75% agarose dye-free cassette and S1 external marker to remove templates smaller than 10 kb. The PacBio Binding Calculator was used to determine conditions to anneal primers to SMRTbells and bind DNA polymerase (P6/C4 chemistry) to form SMRTbell-primer-polymerase complexes. Complexes were bound to Magbeads and washed to remove unbound template and other contaminants. Purified complexes with an added Pacific Biosciences internal control were loaded on a PacBio RS II instrument and run using the MagBead-OCPW protocol. The resulting library was sequenced on 21 SMRT cells with a movie time of 360 min per SMRT cell, totaling ~ 80-fold coverage of the estimated ~ 380 Mb *D. virilis* genome [27].

Assembly of the PacBio reads was performed using Canu v1.5 with default settings [89]. Canu performs read correction on the 40x longest reads based on the genomeSize parameter. This parameter was set to 200 Mb after analyzing the read size distribution to avoid including shorter reads that could result in deterioration of assembly quality. The raw PacBio reads were aligned back to the Canu assembly with pbmm2 v1.0.0. and the assembly was polished with Arrow using gcpp v0.01.e12a6d6. PacBio polishing software was downloaded as part of the pb-assembly metapackage v0.0.6 from bioconda (https://github.com/PacificBiosciences/pb-assembly). A second round of polishing was performed after aligning Illumina reads from strain 160 (SRR1200631) with BWA-MEM v0.7.17-r1188 [90] and correcting errors with Pilon v1.22 [91]. Since *D. virilis* strain 160 is largely colinear with the current *D. virilis* reference genome (strain 15,010–1051.87 [92];, we performed reference-based scaffolding of the strain 160 PacBio assembly using RaGOO v1.1 [93]. As a scaffolding reference, we used the SNP-corrected reference genome for strain 160 [38] (see below) with a single modification consisting of the inclusion of the original scaffold_13050 at the end of chromosome X. This modification was motivated by the recent mapping of two markers present in this scaffold to the base of chromosome X [94]. Assembly metrics were collected with QUAST v5.0.2 (https://github.com/ablab/quast, commit 67a1136, [95]). Assembly completeness was assessed by searching for highly conserved orthologs with BUSCO v3.0.2 [96] using the Diptera ortholog gene set from OrthoDB v9 [97]. Assembly statistics are available in Additional file 1: Table S8. Coordinates of the mitotic CO clusters (see methods below) were lifted over to the final version of the PacBio assembly using minimap2 2.16-r922 [98].

### Annotation of genome resources

Illumina-based reference genomes for strains 9 and 160 [38] were based on the original Sanger shotgun sequence assembly of *D. virilis* [92]. Due to errors in the original reference assembly, genome region 33,464,439-35,498,860 on Chromosome 2 was excluded and genome region 22,673,797-24,330,628 on Chromosome 5 was placed at position 3,761,384 on the same chromosome. Thus, previous strain-specific reference genomes [38] were adjusted for two mis-assemblies and updated as ‘_2’ (strain 9 and strain 160 genomes are available on Figshare at 10.6084/m9.figshare.11803881.v1 or upon request). The genomes were annotated with the most up-to-date gff file for *D. virilis* (v1.6 Flybase, [99]) using Maker v3.31.9 on default settings [100]. TE annotations for Illumina-based reference genomes were obtained using RepeatMasker v4.06 [101] with the custom TE library from Erwin et al. (2015) [38]. TE annotation of the strain 160 PacBio assembly was also obtained using RepeatMasker with the custom TE library from Erwin et al. (2015) [38].

### Crossover quantification

Illumina FASTQ files were parsed according to barcode sequences and trimmed by the University of Kansas Genomics Core facility. The FASTQ files were mapped to the Illumina-based reference genomes for strains 9 and 160 using the multiplex shotgun genotyping (MSG: https://github.com/JaneliaSciComp/msg, v0.1) bioinformatic pipeline for identifying reliable markers and determining ancestry at those markers using HMM [58]. Briefly, reads were mapped with BWA aln 0.5.7 to the strain 9 and 160 parental reference _2 files. Output files were used for HMM determination of ancestry along the length of the chromosomal segments (see control file, Additional file 4, for settings). The MSG pipeline provides both ancestry probability calls and CO positions, along with an estimate of the boundaries for CO positions. The 132 BC1 samples with fewer than 10,000 reads (< 0.005X coverage) were discarded from the analysis. Double COs less than 750 kb apart were discarded as these events were considered extremely improbable. We observed that reads mapping to regions near the telomere and centromere often predicted the same genotype across all samples. In principle, this could be driven by segregation distortion. However, these regions also showed low density for uniquely mapped reads. In addition, segregation distortion for these regions would drive distortion of linked flanking markers, which we did not see. Therefore, we considered these regions problematic and removed them from the analysis. Specifically, COs located within 500 kb of the telomere of the X and 4th chromosome and COs within 700 kb on the 2nd, 3rd, and 5th chromosomes were removed. COs near the proximal edge of our assembly in problematic regions were also removed as follows: within 3.5 Mb on the X chromosome, within 1.1 Mb on the 2nd chromosome, within 1.5 Mb on the 3rd chromosome, within 2.4 Mb on the 4th chromosome, and within 2.3 Mb on the 5th chromosome. The 6th chromosome (corresponding to the 4th in *D. melanogaster*) was also removed from analysis. In addition, we performed some additional curation of COs to remove calls that appeared incorrect. In particular, we removed double COs that were spaced closely in samples with low numbers of reads and ancestry probabilities that were less than 0.9 since these were likely errors from the bioinformatic pipeline. Overall, we favored removing problematic regions from the analysis rather than retaining them. While this limited our analysis by excluding regions of low complexity, this approach is conservative.

### Data analysis

CO outputs from the MSG pipeline were analyzed with R Version 3.4.2 (R Core Team 2017). Ancestry probability outputs were used in the R/qtl package [102] to construct genetic maps. Additional tetrad and interference analyses results are included separately in Additional file 5. We used the lme4 [68] and lsmeans [103] packages for mixed-model testing of CO events in BC1 progeny. The model construction was performed using the glmer() and glm() functions to test the random effects of F1 female and fecundity of the F1 female and the fixed effects of dysgenesis and batches. Fecundity estimates obtained from dysgenic crosses in the second experiment were first used to determine if fecundity had an effect on total CO count. We found that fecundity had no effect on CO count (χ^2^_1_ = 2.02, *p* = 0.155) and excluded it from the final model.

The model for how these effects predict total CO numbers in R was as follows:

$$ \mathrm{glmer}\ \left(\mathrm{CO}.\mathrm{sum}\sim \mathrm{batch}\ \left(\mathrm{fixed}\right)\ast \mathrm{dys}.\mathrm{nondys}\ \left(\mathrm{fixed}\right)\ast \mathrm{parent}\ \left(\mathrm{random}\right),\mathrm{family}=\mathrm{poisson}\ \left(\mathrm{link}=\log \right)\right) $$

We used likelihood ratio tests to determine the significance of each effect on variance in total CO number. We used the Biostrings R package [104] to analyze genomic sequences for correlations between genomic features and recombination. Figures were constructed using ggplot2 [105].

### Analysis of mitotic recombination

Mitotic (or pre-meiotic) recombination is identified by the presence of crossovers that are common among the progeny of a single parent. These are commonly designated as recombination clusters and are distinct from hotspots because they are found only within cohorts of siblings. We used strict criteria to call clusters of recombination at the risk of missing possible clusters for two reasons; COs in our experiment were identified using a shallow sequencing approach which can lead to error in calling CO position and dysgenic females often produce small cohorts further decreasing the probability of observing clusters. Clusters indicating germline mitotic recombination were identified as COs in four or more progeny of a single F1 mother within a 100 kb span; the probability of observing four or more COs in different progeny within a 100 kb span along a 25 Mb chromosome is less than 1E-04 depending on cohort size. Since the fecundity effects of hybrid dysgenesis are highly variable, there was an imbalance in progeny counts from dysgenic and non-dysgenic backcrosses. It was therefore necessary to account for this variation in the estimation of rates of mitotic recombination. This was achieved using a likelihood approach to determine if rates of mitotic cluster formation (α) within the germline and the frequency of mitotic recombination within cohorts (β, conditional upon cluster formation) differed between dysgenic and non-dysgenic parents. Only one mitotic cluster was ever observed per single chromosome so rate estimate was performed on a per chromosome basis. The probability of not observing a cluster event (on a given chromosome) is given by the probability that a mitotic recombination event does not occur in the germline (1-α) plus the probability that a mitotic recombination event does occur (α) but is not observed among the sampled progeny:

$$ {P}_{ClusterNotObs}=\left(1-\alpha \right)+\alpha \times {P}_{ClusterNotSampled\mid ClusterOccurred} $$

Conditional on mitotic recombination occurring, the probability that it was not observed is equal to the probability that three or fewer progeny within the cohort inherit the recombinant chromatid from the mother. This is given with the binomial probability distribution where β is the frequency of recombinant chromosomes transmitted by the mother with the mitotic event:

$$ {P}_{ClusterNotSampled\mid ClusterOccurred}=\sum \limits_{x=0}^3\frac{N!}{x!\left(N-x\right)!}{\beta}^x{\left(1-\beta \right)}^{N-x} $$

where *N* is the total number of progeny in the cohort and β is the frequency of progeny that inherit the recombinant chromosome. Therefore, parents with three or fewer progeny have *P*_*ClusterNotSampled* ∣ *ClusterOccurred*_ = 1 under our criteria.

When a cluster event is observed, the probability of *x* progeny with the recombinant chromosome is given by:

$$ P(x)=\frac{N!}{x!\left(N-x\right)!}{\beta}^x{\left(1-\beta \right)}^{N-x} $$

Overall, the probability that a cluster is observed at a given frequency within a cohort is equal to the probability that mitotic recombination happened (α) multiplied by the probability that it is observed at a given frequency, conditional on it having happened:

$$ {P}_{ClusterObserved}=\alpha \times \frac{N!}{x!\left(N-x\right)!}{\beta}^x{\left(1-\beta \right)}^{N-x} $$

The full likelihood of the data is thus given by:

$$ L(Data)=\prod \limits_{i=1}^m{P}_{i. ClusterNotObserved}\prod \limits_{j=1}^n{P}_{j. ClusterObserved} $$

where *i* is index of mothers without an observed mitotic cluster and *j* as the index of mothers whose progeny indicate a mitotic cluster. Taking the logarithm of our likelihood equation gives

$$ \log \left(L(Data)\right)=\sum \limits_{i=1}^m{P}_{i. ClusterNotObserved}+\sum \limits_{j=1}^n{P}_{j. ClusterObserved} $$

Mitotic recombination was only ever observed on the X and 3rd chromosomes so a combined rate was only estimated for these two chromosomes. To estimate rates of mitotic recombination across dysgenic and non-dysgenic females, we used the Python module Scipy to maximize the log-likelihood of the data based on α and β. Nested likelihood ratio tests were used to determine whether there was support for unique values of α or β in dysgenic or non-dysgenic females. Two three-parameter models were used with distinct cluster formation rates for dysgenic (Dys) and non-dysgenic (NonDys) females (α_Dys_, α_NonDys_, β) and, reciprocally, separate frequencies of transmission of the recombinant chromatid (α, β_Dys_, β_NonDys_). We also used as a four-parameter model with individual estimates for the dysgenic and non-dysgenic mothers (α_Dys_, α_NonDys_, β_Dys_, β_NonDys_). 95% confidence intervals for parameter estimates were obtained by determining parameter values with likelihood scores two log-likelihood units from the ML estimate with other maximum likelihood estimated parameters fixed. We tested if models were significantly improved with the inclusion of additional parameters with a likelihood ratio test (LRT) and a chi-squared distribution with one degree of freedom for every additional parameter estimated. The Python script for the maximum likelihood analysis of the mitotic recombination rates is in Additional file 6. All crossover data used for analysis in this study is included in Additional files 7, 8 and 9.

## Supplementary information

**Additional file 1: Table S1.** Correlations between recombination rates of dysgenic and non-dysgenic flies and high fecund and low fecund dysgenic flies in 250 kb intervals. **Table S2.** Maximum likelihood parameter estimates and model comparisons. **Table S3.** Sampling of BC1 progeny from F1 females in our experiment. **Table S4.** FC1 bar-coded primers and FC2 primer used for PCR amplification for multiplex shotgun sequencing of the pilot batch in this study. **Table S5.** Barcodes used for demultiplexing the pilot batch of BC1 progeny in this study. **Table S6.** i7 primers used for Tn5 tagging and PCR amplification for multiplex shotgun sequencing in the second batch of this study. **Table S7.** i5 primers used for Tn5 tagging and PCR amplification for multiplex shotgun sequencing. **Table S8.** PacBio assembly statistics for *D. virilis* strain 160. **Figure S1.** Correlations between recombination rate and A) SNP Density and B) TE density with and without non-recombining regions. **Figure S2.** Haplotypes of additional BC1 progeny from single F1 mothers with clusters of recombination and potential mitotic recombination events. **Figure S3.** Marey maps of all the *D. virilis* chromosomes without clusters of recombination.

**Additional file 2:** A summary csv table of all intact copies of *Polyphemus*, *Paris*, *Helena*, and *Penelope* in the strain 160 PacBio assembly. Intact copies are defined as less than 5% diverged from the transposable element consensus sequence and having greater than 80% of the length of the consensus sequence.

**Additional file 3:** Sequencing outputs for demultiplexed BC1 progeny.

**Additional file 4:** Example control file used for multiplex shotgun genotyping protocol from Andolfatto et al. 2011.

**Additional file 5:** Interference and tetrad analysis in *Drosophila virilis.*

**Additional file 6:** Python script for maximum likelihood analysis of mitotic recombination rates.

**Additional file 7:** A summary csv table of crossover positions for each BC1 sample with F1 parent, batch number, dysgenic or non-dysgenic, and fecundity. A crossover position ‘0’ means no crossover was observed for a chromosome. Reference position coordinates are based on the s9_2.fasta and s160_2.fasta genome assemblies available on FigShare at https://doi.org/10.6084/m9.figshare.11803881.v1. 

**Additional file 8:** A summary csv table of total crossover number for each BC1 sample with F1 parent, batch number, dysgenic or non-dysgenic, fecundity, and number of BC1 progeny per F1 parent.

**Additional file 9:** A summary csv table of genotype along each chromosome for all BC1 progeny. This csv file can be imported into R/qtl for genetic map construction, crossover interference quantification, and segregation distortion analysis.

## Data Availability

All of the de-multiplexed Illumina sequencing reads from BC1 progeny, PacBio reads for strain 160, and the strain 160 PacBio assembly generated in this study are available at the National Center for Biotechnology Information under accession PRJNA553533. Supplemental files are available at FigShare or upon request.

## Abbreviations

BC1: Backcross Progeny
cM: centimorgan
CO: Crossover
Dys: Dysgenic
DSB: Double-Stranded Break
GSC: Germline Stem Cell
HMM: Hidden Markov Model
LOH: Loss-of-Heterozygosity
LRT: Likelihood Ratio Test
MSG: Multiplexed Shotgun Genotyping
NonDys: Non-Dysgenic
piRNA: PIWI-Interacting RNA
TE: Transposable Element
