# Supplementary material for: Hybrid dysgenesis in Drosophila virilis results in clusters of mitotic recombination and loss-of-heterozygosity but leaves meiotic recombination unaltered
Source: Mob DNA. 2020 Feb 15;11:10. doi: 10.1186/s13100-020-0205-0 (PMC7023781; doi:10.1186/s13100-020-0205-0)

**Table S1:** Correlations between recombination rates of dysgenic and non-dysgenic flies and high fecund and low fecund dysgenic flies in 250 kb intervals. Pearson’s correlation coefficients (R) and significance (*p*-value) are listed. All correlations are significant.

| **Comparison** | | **Chromosome** | | | | | **Total** | **Total minus**  **zero CO** |
| --- | --- | --- | --- | --- | --- | --- | --- | --- |
|  |  | **X** | **2** | **3** | **4** | **5** |  |  |
| Dysgenic vs Non-Dys | R | 0.49 | 0.68 | 0.58 | 0.67 | 0.70 | 0.63 | 0.43 |
|  | *p* | 3.24E-09 | 1.11E-18 | 3.91E-13 | 2.64E-18 | 2.41E-20 | 7.16E-72 | 2.59E-24 |
| High fecund vs low fecund | R | 0.51 | 0.44 | 0.55 | 0.68 | 0.67 | 0.57 | 0.41 |
|  | *p* | 6.97E-10 | 1.76E-07 | 1.83E-11 | 9.60E-19 | 2.27E-18 | 9.25E-57 | 2.70E-20 |

**Table S2:** Maximum likelihood parameter estimates and model comparisons (degrees of freedom = 1).

| Model | Description | Parameter estimates | | *ln* (L) | Model Comparison | *p* value^a^ |
| --- | --- | --- | --- | --- | --- | --- |
|  |  | α (95% CIs)^b^ | β (95% CIs) |  |  |  |
| 1 | α and β all F1 mothers | 0.084 (0.038, 0.15) | 0.67 (0.60, 0.74) | -81.7 |  |  |
| 2 | Separate α for dys and non-dys | Dys: 0.12 (0.054, 0.23)  Non: 0.026 (0.00, 0.11) | 0.67 (0.59, 0.74) | -80.1 | M1 vs M2 | 0.066 |
| 3 | Separate β for dys and non-dys | 0.12 (0.057, 0.22) | Dys: 0.78 (0.71, 0.85)  Non: 0.11 (0.038, 0.23) | -56.0 | M1 vs M3 | 6.9E-13 |
| 4 | Separate α and β for dys and non-dys | Dys: 0.11  Non: > 1.0 | Dys: 0.78  Non: 0.066 | -55.0 | M3 vs M4 | 0.17 |

^a^ Likelihood ratio test assuming a χ^2^ distribution

^b^ 95% CIs were not calculated for Model 4, parameter estimations were over-dispersed and not contained within reasonable boundaries.

**Table S3:** Sampling of BC1 progeny from F1 females in our experiment. Dysgenic F1 females were categorized as low or high fecundity based on the number of total progeny produced.

| F1 Parent Identifier | Dys or  Non-Dys | Batch  Number | Low or High Fecundity | Progeny collected F1  < 10 days old | Progeny  collected F1 >10 days old | Total  Progeny Collected |
| --- | --- | --- | --- | --- | --- | --- |
| 101 | Dysgenic | 1 | low | 2 | 0 | 2 |
| 102 | Non-dys | 1 | NA | 11 | 0 | 11 |
| 111 | Dysgenic | 1 | low | 10 | 0 | 10 |
| 112 | Non-dys | 1 | NA | 9 | 0 | 9 |
| 122 | Non-dys | 1 | NA | 11 | 0 | 11 |
| 132 | Non-dys | 1 | NA | 9 | 0 | 9 |
| 142 | Non-dys | 1 | NA | 8 | 0 | 8 |
| 211 | Dysgenic | 1 | low | 2 | 0 | 2 |
| 221 | Dysgenic | 1 | low | 3 | 0 | 3 |
| 301 | Dysgenic | 1 | low | 16 | 0 | 16 |
| 601 | Dysgenic | 1 | low | 5 | 0 | 5 |
| 701 | Dysgenic | 1 | high | 49 | 0 | 49 |
| 702 | Non-dys | 1 | NA | 10 | 0 | 10 |
| 801 | Dysgenic | 1 | low | 3 | 0 | 3 |
| 802 | Non-dys | 1 | NA | 9 | 0 | 9 |
| 902 | Non-dys | 1 | NA | 9 | 0 | 9 |
| 1 | Non-dys | 2 | NA | 13 | 0 | 13 |
| 2 | Non-dys | 2 | NA | 8 | 0 | 8 |
| 3 | Non-dys | 2 | NA | 29 | 0 | 29 |
| 5 | Non-dys | 2 | NA | 19 | 0 | 19 |
| 8 | Non-dys | 2 | NA | 14 | 0 | 14 |
| 9 | Non-dys | 2 | NA | 9 | 0 | 9 |
| 10 | Non-dys | 2 | NA | 12 | 0 | 12 |
| 12 | Non-dys | 2 | NA | 20 | 0 | 20 |
| 14 | Non-dys | 2 | NA | 12 | 0 | 12 |
| 15 | Non-dys | 2 | NA | 17 | 0 | 17 |
| 16 | Non-dys | 2 | NA | 20 | 0 | 20 |
| 20 | Non-dys | 2 | NA | 26 | 0 | 26 |
| 4003 | Dysgenic | 2 | low | 0 | 3 | 3 |
| 4004 | Dysgenic | 2 | low | 2 | 2 | 4 |
| 4006 | Dysgenic | 2 | low | 2 | 0 | 2 |
| 4010 | Dysgenic | 2 | low | 2 | 8 | 10 |
| 4012 | Dysgenic | 2 | low | 7 | 7 | 14 |
| 4013 | Dysgenic | 2 | low | 9 | 4 | 13 |
| 4016 | Dysgenic | 2 | high | 24 | 0 | 24 |
| 4018 | Dysgenic | 2 | low | 2 | 0 | 2 |
| 4019 | Dysgenic | 2 | low | 14 | 0 | 14 |
| 4021 | Dysgenic | 2 | low | 2 | 0 | 2 |
| 4022 | Dysgenic | 2 | low | 1 | 0 | 1 |
| 4027 | Dysgenic | 2 | low | 2 | 0 | 2 |
| 4029 | Dysgenic | 2 | high | 32 | 0 | 32 |
| 4031 | Dysgenic | 2 | low | 5 | 0 | 5 |
| 4040 | Dysgenic | 2 | low | 1 | 0 | 1 |
| 4050 | Dysgenic | 2 | low | 1 | 0 | 1 |
| 4065 | Dysgenic | 2 | low | 0 | 1 | 1 |
| 4070 | Dysgenic | 2 | low | 1 | 0 | 1 |
| 4071 | Dysgenic | 2 | low | 1 | 0 | 1 |
| 4078 | Dysgenic | 2 | low | 5 | 0 | 5 |
| 4084 | Dysgenic | 2 | low | 11 | 0 | 11 |
| 4086 | Dysgenic | 2 | low | 4 | 0 | 4 |
| 4087 | Dysgenic | 2 | low | 1 | 0 | 1 |
| 4092 | Dysgenic | 2 | low | 9 | 1 | 10 |
| 4097 | Dysgenic | 2 | low | 6 | 0 | 6 |
| 4099 | Dysgenic | 2 | low | 2 | 1 | 3 |
| 4100 | Dysgenic | 2 | low | 11 | 2 | 13 |
| 4101 | Dysgenic | 2 | low | 6 | 4 | 10 |
| 4102 | Dysgenic | 2 | low | 2 | 0 | 2 |
| 4104 | Dysgenic | 2 | low | 9 | 0 | 9 |
| 4108 | Dysgenic | 2 | low | 6 | 0 | 6 |
| 4109 | Dysgenic | 2 | low | 1 | 0 | 1 |
| 4120 | Dysgenic | 2 | low | 1 | 0 | 1 |
| 4133 | Dysgenic | 2 | low | 0 | 1 | 1 |
| 4144 | Dysgenic | 2 | low | 0 | 1 | 1 |
| 4147 | Dysgenic | 2 | low | 2 | 1 | 3 |
| 5001 | Dysgenic | 2 | low | 9 | 1 | 10 |
| 5002 | Dysgenic | 2 | low | 2 | 0 | 2 |
| 5006 | Dysgenic | 2 | low | 1 | 0 | 1 |
| 5007 | Dysgenic | 2 | low | 1 | 1 | 2 |
| 5010 | Dysgenic | 2 | low | 2 | 1 | 3 |
| 5011 | Dysgenic | 2 | high | 32 | 0 | 32 |
| 5016 | Dysgenic | 2 | low | 2 | 0 | 2 |
| 5017 | Dysgenic | 2 | low | 6 | 0 | 6 |
| 5019 | Dysgenic | 2 | high | 30 | 0 | 30 |
| 5020 | Dysgenic | 2 | low | 2 | 0 | 2 |
| 5022 | Dysgenic | 2 | low | 10 | 1 | 11 |
| 5023 | Dysgenic | 2 | low | 0 | 1 | 1 |
| 5024 | Dysgenic | 2 | low | 4 | 0 | 4 |
| 5025 | Dysgenic | 2 | low | 1 | 0 | 1 |
| 5026 | Dysgenic | 2 | low | 1 | 0 | 1 |
| 5027 | Dysgenic | 2 | high | 35 | 0 | 35 |
| 5033 | Dysgenic | 2 | low | 0 | 1 | 1 |
| 5040 | Dysgenic | 2 | low | 1 | 0 | 1 |
| 5066 | Dysgenic | 2 | low | 2 | 0 | 2 |
| 5089 | Dysgenic | 2 | low | 8 | 3 | 11 |
| 5092 | Dysgenic | 2 | low | 3 | 0 | 3 |
| 5095 | Dysgenic | 2 | low | 3 | 1 | 4 |
| 5096 | Dysgenic | 2 | high | 40 | 0 | 40 |
| 5097 | Dysgenic | 2 | low | 4 | 0 | 4 |
| 5100 | Dysgenic | 2 | low | 6 | 2 | 8 |
| 5104 | Dysgenic | 2 | low | 10 | 0 | 10 |
| 5105 | Dysgenic | 2 | low | 3 | 1 | 4 |
| 5108 | Dysgenic | 2 | low | 0 | 2 | 2 |
| 5111 | Dysgenic | 2 | low | 8 | 2 | 10 |

**Table S4:** FC1 bar-coded primers and FC2 primer used for PCR amplification for multiplex shotgun sequencing of the pilot batch in this study.

| **NAME** | **FC1 Index Barcode** | **PRIMER (5'-3')** | **FC1 Demultiplex Orientation** |
| --- | --- | --- | --- |
| FC1_PCR_Index5 | CACTGT | CAAGCAGAAGACGGCATACGAGATCACTGTGTGACTGGAGTTCAGACGTGTGCTC*T | ACAGTG |
| FC1_PCR_Index19 | TTTCAC | CAAGCAGAAGACGGCATACGAGATTTTCACGTGACTGGAGTTCAGACGTGTGCTC*T | GTCAAA |
| FC2_PCR_Primer | -- | AATGATACGGCGACCACCGAGATCTACACTCTTTCCCTACACGACGCTCTTCCGATC*T | -- |

**Table S5:** Barcodes used for demultiplexing the pilot batch of BC1 progeny in this study. The barcodes are a part of the FC1 and FC2 oligonucleotide adaptors. The FC2 oligonucleotide sequence is ACACTCTTTCCCTACACGACGCTCTTCCGANNNNNN where NNNNNN is one of the six base-pair barcodes and the FC1 oligonucleotide sequence is p-TAnnnnnnTCGGA

AGAGCTCGTATGCCGTCTTCTGCTTG where p is a phosphate and nnnnnn is the reverse complement of the corresponding FC2 barcode.

| CGTCTG | CTCTAC | GCGCGT | AAGGAC | TATATG | GGCATT | CAATAT | GATGAG |
| --- | --- | --- | --- | --- | --- | --- | --- |
| TCTCGT | CATAGC | CTTGCG | ATATGG | GGTTAC | AAGCGA | AAACGG | TCTATT |
| CCCTAT | TCCACG | GGGAAT | TTCCTG | TAGTCG | CACTGA | GAGAGT | GGAATG |
| AGGCGT | GTTCAG | ACTGTT | GACAAT | CGATAC | ATCACG | GTCGAT | GATCTG |
| CTCGGT | ACGTAC | CTAACG | GCGTAT | TTAGCG | ACCGAT | TAAGCA | GAATAC |
| ATTCTG | GCTGAT | CTGACA | TAGACA | GGAAGT | TATAGT | AGCTCA | CTCTGG |
| TGAATT | CAACTG | ATGATG | GTCCGG | GCACAG | ATCCAG | GAATGG | TGTAAT |
| TCTCTG | AAATAG | AACCTG | TAATTG | GAGTAG | AATCCG | GTAACA | AAGCAG |
| AATGCC | TTACTT | AAGGTT | AGAGTT | AGTCAC | CAGGTG | ATCTTG | GGACCA |
| TTTCAT | CATTGT | AACTCG | AATAAG | GGGTTG | CCACCA | GTTGCA | CCCACA |
| GTCCTT | GCCGTG | GACACG | TGACTG | GCCTGG | TCGAGT | ATACCG | GGCCGT |
| GAAGTG | AGGCTG | CGCTTG | CTTCGG | AATGTG | GGCCTG | ATAGGT | AATGGT |

**Table S6:** i7 primers used for Tn5 tagging and PCR amplification for multiplex shotgun sequencing in the second batch of this study.

| **NAME** | **i7 Index Barcode** | **PRIMER (5'-3')** | **i7 Demultiplex Orientation** |
| --- | --- | --- | --- |
| **jpbhb701** | AACGTGAT | CAAGCAGAAGACGGCATACGAGATAACGTGATGTCTCGTGGGCTCGG | ATCACGTT |
| **jpbhb702** | AAACATCG | CAAGCAGAAGACGGCATACGAGATAAACATCGGTCTCGTGGGCTCGG | CGATGTTT |
| **jpbhb703** | ATGCCTAA | CAAGCAGAAGACGGCATACGAGATATGCCTAAGTCTCGTGGGCTCGG | TTAGGCAT |
| **jpbhb704** | AGTGGTCA | CAAGCAGAAGACGGCATACGAGATAGTGGTCAGTCTCGTGGGCTCGG | TGACCACT |
| **jpbhb705** | ACCACTGT | CAAGCAGAAGACGGCATACGAGATACCACTGTGTCTCGTGGGCTCGG | ACAGTGGT |
| **jpbhb706** | ACATTGGC | CAAGCAGAAGACGGCATACGAGATACATTGGCGTCTCGTGGGCTCGG | GCCAATGT |
| **jpbhb707** | CAGATCTG | CAAGCAGAAGACGGCATACGAGATCAGATCTGGTCTCGTGGGCTCGG | CAGATCTG |
| **jpbhb708** | CATCAAGT | CAAGCAGAAGACGGCATACGAGATCATCAAGTGTCTCGTGGGCTCGG | ACTTGATG |
| **jpbhb709** | CGCTGATC | CAAGCAGAAGACGGCATACGAGATCGCTGATCGTCTCGTGGGCTCGG | GATCAGCG |
| **jpbhb710** | ACAAGCTA | CAAGCAGAAGACGGCATACGAGATACAAGCTAGTCTCGTGGGCTCGG | TAGCTTGT |
| **jpbhb711** | CTGTAGCC | CAAGCAGAAGACGGCATACGAGATCTGTAGCCGTCTCGTGGGCTCGG | GGCTACAG |
| **jpbhb712** | AGTACAAG | CAAGCAGAAGACGGCATACGAGATAGTACAAGGTCTCGTGGGCTCGG | CTTGTACT |
| **jpbhb713** | AACAACCA | CAAGCAGAAGACGGCATACGAGATAACAACCAGTCTCGTGGGCTCGG | TGGTTGTT |
| **jpbhb714** | AACCGAGA | CAAGCAGAAGACGGCATACGAGATAACCGAGAGTCTCGTGGGCTCGG | TCTCGGTT |
| **jpbhb715** | AACGCTTA | CAAGCAGAAGACGGCATACGAGATAACGCTTAGTCTCGTGGGCTCGG | TAAGCGTT |
| **jpbhb716** | AAGACGGA | CAAGCAGAAGACGGCATACGAGATAAGACGGAGTCTCGTGGGCTCGG | TCCGTCTT |
| **jpbhb717** | AAGGTACA | CAAGCAGAAGACGGCATACGAGATAAGGTACAGTCTCGTGGGCTCGG | TGTACCTT |
| **jpbhb718** | ACACAGAA | CAAGCAGAAGACGGCATACGAGATACACAGAAGTCTCGTGGGCTCGG | TTCTGTGT |
| **jpbhb719** | ACAGCAGA | CAAGCAGAAGACGGCATACGAGATACAGCAGAGTCTCGTGGGCTCGG | TCTGCTGT |
| **jpbhb720** | ACCTCCAA | CAAGCAGAAGACGGCATACGAGATACCTCCAAGTCTCGTGGGCTCGG | TTGGAGGT |
| **jpbhb721** | ACGCTCGA | CAAGCAGAAGACGGCATACGAGATACGCTCGAGTCTCGTGGGCTCGG | TCGAGCGT |
| **jpbhb722** | ACGTATCA | CAAGCAGAAGACGGCATACGAGATACGTATCAGTCTCGTGGGCTCGG | TGATACGT |
| **jpbhb723** | ACTATGCA | CAAGCAGAAGACGGCATACGAGATACTATGCAGTCTCGTGGGCTCGG | TGCATAGT |
| **jpbhb724** | AGAGTCAA | CAAGCAGAAGACGGCATACGAGATAGAGTCAAGTCTCGTGGGCTCGG | TTGACTCT |
| **jpbhb725** | AGATCGCA | CAAGCAGAAGACGGCATACGAGATAGATCGCAGTCTCGTGGGCTCGG | TGCGATCT |
| **jpbhb726** | AGCAGGAA | CAAGCAGAAGACGGCATACGAGATAGCAGGAAGTCTCGTGGGCTCGG | TTCCTGCT |
| **jpbhb727** | AGTCACTA | CAAGCAGAAGACGGCATACGAGATAGTCACTAGTCTCGTGGGCTCGG | TAGTGACT |
| **jpbhb728** | ATCCTGTA | CAAGCAGAAGACGGCATACGAGATATCCTGTAGTCTCGTGGGCTCGG | TACAGGAT |
| **jpbhb729** | ATTGAGGA | CAAGCAGAAGACGGCATACGAGATATTGAGGAGTCTCGTGGGCTCGG | TCCTCAAT |
| **jpbhb730** | CAACCACA | CAAGCAGAAGACGGCATACGAGATCAACCACAGTCTCGTGGGCTCGG | TGTGGTTG |
| **jpbhb731** | GACTAGTA | CAAGCAGAAGACGGCATACGAGATGACTAGTAGTCTCGTGGGCTCGG | TACTAGTC |
| **jpbhb732** | CAATGGAA | CAAGCAGAAGACGGCATACGAGATCAATGGAAGTCTCGTGGGCTCGG | TTCCATTG |
| **jpbhb733** | CACTTCGA | CAAGCAGAAGACGGCATACGAGATCACTTCGAGTCTCGTGGGCTCGG | TCGAAGTG |
| **jpbhb734** | CAGCGTTA | CAAGCAGAAGACGGCATACGAGATCAGCGTTAGTCTCGTGGGCTCGG | TAACGCTG |
| **jpbhb735** | CATACCAA | CAAGCAGAAGACGGCATACGAGATCATACCAAGTCTCGTGGGCTCGG | TTGGTATG |
| **jpbhb736** | CCAGTTCA | CAAGCAGAAGACGGCATACGAGATCCAGTTCAGTCTCGTGGGCTCGG | TGAACTGG |
| **jpbhb737** | CCGAAGTA | CAAGCAGAAGACGGCATACGAGATCCGAAGTAGTCTCGTGGGCTCGG | TACTTCGG |
| **jpbhb738** | CCGTGAGA | CAAGCAGAAGACGGCATACGAGATCCGTGAGAGTCTCGTGGGCTCGG | TCTCACGG |
| **jpbhb739** | CCTCCTGA | CAAGCAGAAGACGGCATACGAGATCCTCCTGAGTCTCGTGGGCTCGG | TCAGGAGG |
| **jpbhb740** | CGAACTTA | CAAGCAGAAGACGGCATACGAGATCGAACTTAGTCTCGTGGGCTCGG | TAAGTTCG |
| **jpbhb741** | CGACTGGA | CAAGCAGAAGACGGCATACGAGATCGACTGGAGTCTCGTGGGCTCGG | TCCAGTCG |
| **jpbhb742** | CGCATACA | CAAGCAGAAGACGGCATACGAGATCGCATACAGTCTCGTGGGCTCGG | TGTATGCG |
| **jpbhb743** | CTCAATGA | CAAGCAGAAGACGGCATACGAGATCTCAATGAGTCTCGTGGGCTCGG | TCATTGAG |
| **jpbhb744** | CTGAGCCA | CAAGCAGAAGACGGCATACGAGATCTGAGCCAGTCTCGTGGGCTCGG | TGGCTCAG |
| **jpbhb745** | CTGGCATA | CAAGCAGAAGACGGCATACGAGATCTGGCATAGTCTCGTGGGCTCGG | TATGCCAG |
| **jpbhb746** | GAATCTGA | CAAGCAGAAGACGGCATACGAGATGAATCTGAGTCTCGTGGGCTCGG | TCAGATTC |
| **jpbhb747** | CAAGACTA | CAAGCAGAAGACGGCATACGAGATCAAGACTAGTCTCGTGGGCTCGG | TAGTCTTG |
| **jpbhb748** | GAGCTGAA | CAAGCAGAAGACGGCATACGAGATGAGCTGAAGTCTCGTGGGCTCGG | TTCAGCTC |
| **jpbhb749** | GATAGACA | CAAGCAGAAGACGGCATACGAGATGATAGACAGTCTCGTGGGCTCGG | TGTCTATC |
| **jpbhb750** | GCCACATA | CAAGCAGAAGACGGCATACGAGATGCCACATAGTCTCGTGGGCTCGG | TATGTGGC |
| **jpbhb751** | GCGAGTAA | CAAGCAGAAGACGGCATACGAGATGCGAGTAAGTCTCGTGGGCTCGG | TTACTCGC |
| **jpbhb752** | GCTAACGA | CAAGCAGAAGACGGCATACGAGATGCTAACGAGTCTCGTGGGCTCGG | TCGTTAGC |
| **jpbhb753** | GCTCGGTA | CAAGCAGAAGACGGCATACGAGATGCTCGGTAGTCTCGTGGGCTCGG | TACCGAGC |
| **jpbhb754** | GGAGAACA | CAAGCAGAAGACGGCATACGAGATGGAGAACAGTCTCGTGGGCTCGG | TGTTCTCC |
| **jpbhb755** | GGTGCGAA | CAAGCAGAAGACGGCATACGAGATGGTGCGAAGTCTCGTGGGCTCGG | TTCGCACC |
| **jpbhb756** | GTACGCAA | CAAGCAGAAGACGGCATACGAGATGTACGCAAGTCTCGTGGGCTCGG | TTGCGTAC |
| **jpbhb757** | GTCGTAGA | CAAGCAGAAGACGGCATACGAGATGTCGTAGAGTCTCGTGGGCTCGG | TCTACGAC |
| **jpbhb758** | GTCTGTCA | CAAGCAGAAGACGGCATACGAGATGTCTGTCAGTCTCGTGGGCTCGG | TGACAGAC |
| **jpbhb759** | GTGTTCTA | CAAGCAGAAGACGGCATACGAGATGTGTTCTAGTCTCGTGGGCTCGG | TAGAACAC |
| **jpbhb760** | TAGGATGA | CAAGCAGAAGACGGCATACGAGATTAGGATGAGTCTCGTGGGCTCGG | TCATCCTA |
| **jpbhb761** | TATCAGCA | CAAGCAGAAGACGGCATACGAGATTATCAGCAGTCTCGTGGGCTCGG | TGCTGATA |
| **jpbhb762** | TCCGTCTA | CAAGCAGAAGACGGCATACGAGATTCCGTCTAGTCTCGTGGGCTCGG | TAGACGGA |
| **jpbhb763** | TCTTCACA | CAAGCAGAAGACGGCATACGAGATTCTTCACAGTCTCGTGGGCTCGG | TGTGAAGA |
| **jpbhb764** | TGAAGAGA | CAAGCAGAAGACGGCATACGAGATTGAAGAGAGTCTCGTGGGCTCGG | TCTCTTCA |
| **jpbhb765** | TGGAACAA | CAAGCAGAAGACGGCATACGAGATTGGAACAAGTCTCGTGGGCTCGG | TTGTTCCA |
| **jpbhb766** | TGGCTTCA | CAAGCAGAAGACGGCATACGAGATTGGCTTCAGTCTCGTGGGCTCGG | TGAAGCCA |
| **jpbhb767** | TGGTGGTA | CAAGCAGAAGACGGCATACGAGATTGGTGGTAGTCTCGTGGGCTCGG | TACCACCA |
| **jpbhb768** | TTCACGCA | CAAGCAGAAGACGGCATACGAGATTTCACGCAGTCTCGTGGGCTCGG | TGCGTGAA |
| **jpbhb769** | AACTCACC | CAAGCAGAAGACGGCATACGAGATAACTCACCGTCTCGTGGGCTCGG | GGTGAGTT |
| **jpbhb770** | AAGAGATC | CAAGCAGAAGACGGCATACGAGATAAGAGATCGTCTCGTGGGCTCGG | GATCTCTT |
| **jpbhb771** | AAGGACAC | CAAGCAGAAGACGGCATACGAGATAAGGACACGTCTCGTGGGCTCGG | GTGTCCTT |
| **jpbhb772** | AATCCGTC | CAAGCAGAAGACGGCATACGAGATAATCCGTCGTCTCGTGGGCTCGG | GACGGATT |
| **jpbhb773** | AATGTTGC | CAAGCAGAAGACGGCATACGAGATAATGTTGCGTCTCGTGGGCTCGG | GCAACATT |
| **jpbhb774** | ACACGACC | CAAGCAGAAGACGGCATACGAGATACACGACCGTCTCGTGGGCTCGG | GGTCGTGT |
| **jpbhb775** | ACAGATTC | CAAGCAGAAGACGGCATACGAGATACAGATTCGTCTCGTGGGCTCGG | GAATCTGT |
| **jpbhb776** | AGATGTAC | CAAGCAGAAGACGGCATACGAGATAGATGTACGTCTCGTGGGCTCGG | GTACATCT |
| **jpbhb777** | AGCACCTC | CAAGCAGAAGACGGCATACGAGATAGCACCTCGTCTCGTGGGCTCGG | GAGGTGCT |
| **jpbhb778** | AGCCATGC | CAAGCAGAAGACGGCATACGAGATAGCCATGCGTCTCGTGGGCTCGG | GCATGGCT |
| **jpbhb779** | AGGCTAAC | CAAGCAGAAGACGGCATACGAGATAGGCTAACGTCTCGTGGGCTCGG | GTTAGCCT |
| **jpbhb780** | ATAGCGAC | CAAGCAGAAGACGGCATACGAGATATAGCGACGTCTCGTGGGCTCGG | GTCGCTAT |
| **jpbhb781** | ATCATTCC | CAAGCAGAAGACGGCATACGAGATATCATTCCGTCTCGTGGGCTCGG | GGAATGAT |
| **jpbhb782** | ATTGGCTC | CAAGCAGAAGACGGCATACGAGATATTGGCTCGTCTCGTGGGCTCGG | GAGCCAAT |
| **jpbhb783** | CAAGGAGC | CAAGCAGAAGACGGCATACGAGATCAAGGAGCGTCTCGTGGGCTCGG | GCTCCTTG |
| **jpbhb784** | CACCTTAC | CAAGCAGAAGACGGCATACGAGATCACCTTACGTCTCGTGGGCTCGG | GTAAGGTG |
| **jpbhb785** | CCATCCTC | CAAGCAGAAGACGGCATACGAGATCCATCCTCGTCTCGTGGGCTCGG | GAGGATGG |
| **jpbhb786** | CCGACAAC | CAAGCAGAAGACGGCATACGAGATCCGACAACGTCTCGTGGGCTCGG | GTTGTCGG |
| **jpbhb787** | CCTAATCC | CAAGCAGAAGACGGCATACGAGATCCTAATCCGTCTCGTGGGCTCGG | GGATTAGG |
| **jpbhb788** | CCTCTATC | CAAGCAGAAGACGGCATACGAGATCCTCTATCGTCTCGTGGGCTCGG | GATAGAGG |
| **jpbhb789** | CGACACAC | CAAGCAGAAGACGGCATACGAGATCGACACACGTCTCGTGGGCTCGG | GTGTGTCG |
| **jpbhb790** | CGGATTGC | CAAGCAGAAGACGGCATACGAGATCGGATTGCGTCTCGTGGGCTCGG | GCAATCCG |
| **jpbhb791** | CTAAGGTC | CAAGCAGAAGACGGCATACGAGATCTAAGGTCGTCTCGTGGGCTCGG | GACCTTAG |
| **jpbhb792** | GAACAGGC | CAAGCAGAAGACGGCATACGAGATGAACAGGCGTCTCGTGGGCTCGG | GCCTGTTC |
| **jpbhb793** | GACAGTGC | CAAGCAGAAGACGGCATACGAGATGACAGTGCGTCTCGTGGGCTCGG | GCACTGTC |
| **jpbhb794** | GAGTTAGC | CAAGCAGAAGACGGCATACGAGATGAGTTAGCGTCTCGTGGGCTCGG | GCTAACTC |
| **jpbhb795** | GATGAATC | CAAGCAGAAGACGGCATACGAGATGATGAATCGTCTCGTGGGCTCGG | GATTCATC |
| **jpbhb796** | GCCAAGAC | CAAGCAGAAGACGGCATACGAGATGCCAAGACGTCTCGTGGGCTCGG | GTCTTGGC |

**Table S7:** i5 primers used for Tn5 tagging and PCR amplification for multiplex shotgun sequencing.

| NAME | i5 Index Barcode | FINAL PRIMER (5'-3') | i5 Demultiplex Orientation |
| --- | --- | --- | --- |
| sjmhb501 | TAGATCGC | AATGATACGGCGACCACCGAGATCTACACTAGATCGCTCGTCGGCAGCGTC | TAGATCGC |
| sjmhb502 | CTCTCTAT | AATGATACGGCGACCACCGAGATCTACACCTCTCTATTCGTCGGCAGCGTC | CTCTCTAT |
| sjmhb503 | TATCCTCT | AATGATACGGCGACCACCGAGATCTACACTATCCTCTTCGTCGGCAGCGTC | TATCCTCT |
| sjmhb504 | AGAGTAGA | AATGATACGGCGACCACCGAGATCTACACAGAGTAGATCGTCGGCAGCGTC | AGAGTAGA |
| sjmhb505 | GTAAGGAG | AATGATACGGCGACCACCGAGATCTACACGTAAGGAGTCGTCGGCAGCGTC | GTAAGGAG |
| sjmhb506 | ACTGCATA | AATGATACGGCGACCACCGAGATCTACACACTGCATATCGTCGGCAGCGTC | ACTGCATA |
| sjmhb507 | AAGGAGTA | AATGATACGGCGACCACCGAGATCTACACAAGGAGTATCGTCGGCAGCGTC | AAGGAGTA |
| sjmhb508 | CTAAGCCT | AATGATACGGCGACCACCGAGATCTACACCTAAGCCTTCGTCGGCAGCGTC | CTAAGCCT |

**Table S8:** PacBio assembly statistics for *D. virilis* strain 160.

| **Statistic** | **Value** |
| --- | --- |
| Length (bp) | 169,775,145 |
| # of contigs | 211 |
| # of scaffolds | 45 |
| Contig N50 (bp) | 5,099,607 |
| Scaffold N50 (bp) | 31,075,311 |
| # of unplaced contigs^*^ | 39 |
| Length of unplaced contigs (bp)^*^ | 4,275,011 |
| BUSCO Complete (%)^**^ | 98.8 |
| Single copy | 98.2 |
| Duplicated | 0.6 |
| BUSCO Fragmented^**^ | 0.6 |
| BUSCO Missing^**^ | 0.6 |

* Unplaced contigs are contigs that could not be assigned to one of the chromosome arms based on alignments to the reference.

** BUSCO score expressed as the percentage of genes within each category. The Diptera ortholog gene set v9 with 2,799 genes was used in the search.

**Figure S1:** Correlations between recombination rate and A) SNP Density and B) TE density with and without non-recombining regions. Density is the percentage of a 250 kb interval made up of either SNPs or TEs. The data is the summation of intervals across all chromosomes.


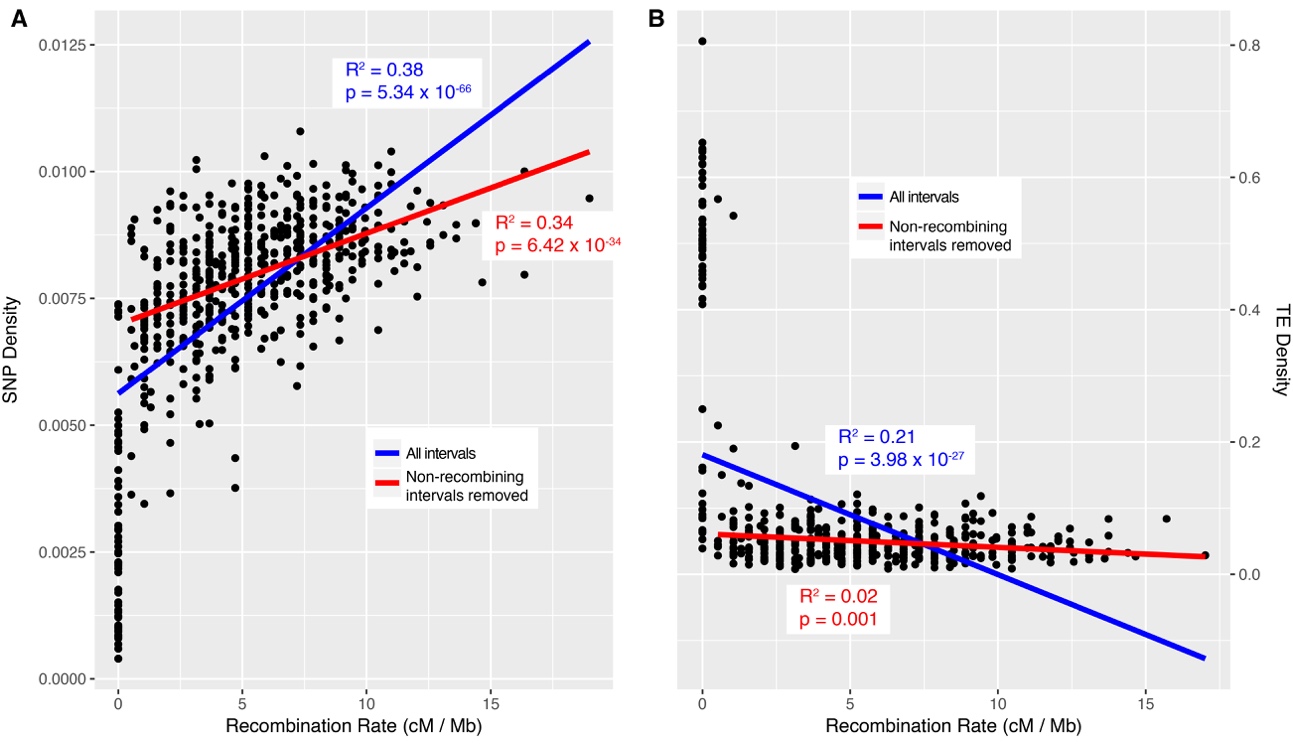


**Figure S2:** Haplotypes of additional BC1 progeny from single F1 mothers with clusters of recombination and potential mitotic recombination events. The haplotypes are from progeny of the following F1 mothers (dysgenic unless specified): A) X chromosome of non-dysgenic 3, B) X chromosome of 4013, C) X chromosome of 5089, D) 3^rd^ chromosome of 5019, E) 3^rd^ chromosome of 111, and F) 3^rd^ chromosome of 5022.


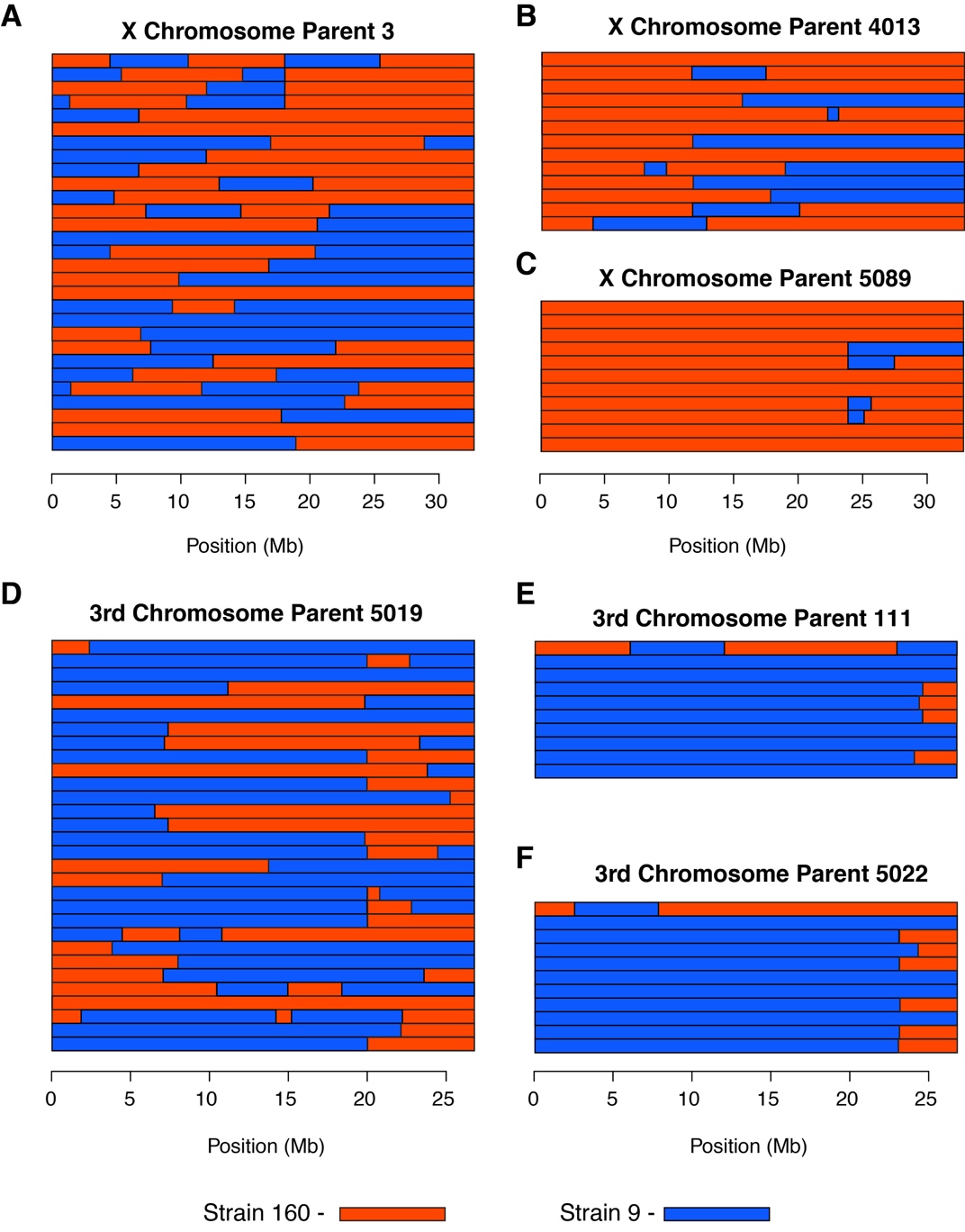


**Figure S3:** Marey maps of all the *D. virilis* chromosomes without clusters of recombination. Changes to the genetic lengths of the chromosomes were minimal, the most significant change was a 5 cM increase of the X chromosome with the removal of progeny with a loss of heterozygosity.


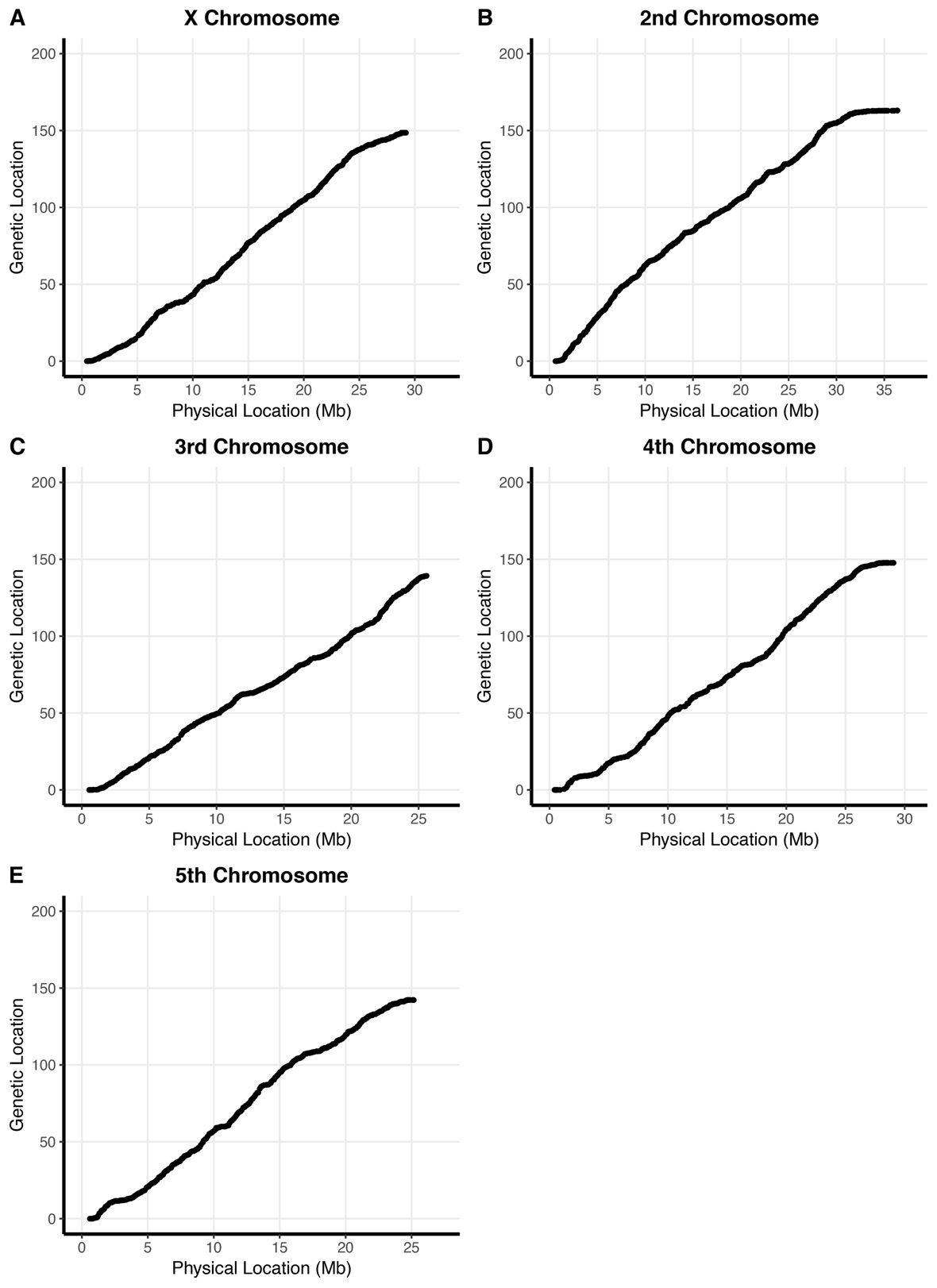

Supplement: Supplementary file 1 — Additional file 1: Table S1. Correlations between recombination rates of dysgenic and non-dysgenic flies and high fecund and low fecund dysgenic flies in 250 kb intervals. Table S2. Maximum likelihood parameter estimates and model comparisons. Table S3. Sampling of BC1 progeny from F1 females in our experiment. Table S4. FC1 bar-coded primers and FC2 primer used for PCR amplification for multiplex shotgun sequencing of the pilot batch in this study. Table S5. Barcodes used for demultiplexing the pilot batch of BC1 progeny in this study. Table S6. i7 primers used for Tn5 tagging and PCR amplification for multiplex shotgun sequencing in the second batch of this study. Table S7. i5 primers used for Tn5 tagging and PCR amplification for multiplex shotgun sequencing. Table S8. PacBio assembly statistics for D. virilis strain 160. Figure S1. Correlations between recombination rate and A) SNP Density and B) TE density with and without non-recombining regions. Figure S2. Haplotypes of additional BC1 progeny from single F1 mothers with clusters of recombination and potential mitotic recombination events. Figure S3. Marey maps of all the D. virilis chromosomes without clusters of recombination. [file 13100_2020_205_MOESM1_ESM.docx]
