# Supplementary material for: Hybrid dysgenesis in Drosophila virilis results in clusters of mitotic recombination and loss-of-heterozygosity but leaves meiotic recombination unaltered
Source: Mob DNA. 2020 Feb 15;11:10. doi: 10.1186/s13100-020-0205-0 (PMC7023781; doi:10.1186/s13100-020-0205-0)

**Interference and tetrad analysis in *Drosophila virilis***

Interference reduces the probability of an additional CO in proximity to other COs. We calculated interference in *D. virilis* using the Housworth-Stahl model to calculate *nu*, a unitless measure of interference, with a maximum likelihood function based on intercrossover distances [1]. If COs are not subject to interference, intercrossover distances are Poisson distributed and *nu* is equal to one [2]. *nu* values greater than one are observed when COs are spaced more evenly than expected under a Poisson process. Each chromosome in *D. virilis* has detectable interference between COs with an average *nu* ~ 3 (Additional File 2: Table S9). The Houseworth-Stahl model also estimates the percentage of COs produced through an alternative pathway not subject to interference as the escape parameter *P* [3]. Less than 1% of the COs in our study are estimated to be produced through the alternative CO pathway (Additional File 2: Table S9). In contrast to interference, assurance is the recombination control mechanism that maintains a minimal number of COs on each chromosome to ensure proper chromosome segregation during meiosis. In the absence of CO assurance and interference, the distribution of COs should resemble a Poisson distribution with variance in CO number equal to the mean. We find the CO mean and variance are not equal (7.3 and 4.9, respectively) and the distribution of CO counts is significantly different from a Poisson distribution (χ^2^_14_ = 53.6, *p* = 5.74E-08). Moreover, *D. virilis* has a higher than expected frequency of individuals with CO numbers close to the mean (5-8 COs) and a lower than expected frequency of extreme CO numbers (Additional File 2: Figure S4). This indicates the collective action of CO assurance and interference.

In many organisms, the total number of COs between markers in a single tetrad is unobservable because COs are typically detected using random spore analysis based on only one of the four chromatids resulting from meiosis. In contrast, tetrad analysis uses the number of COs for each of the four meiotic products to estimate the frequency of non-exchange tetrads (*E*_0_), single-exchange tetrads (*E*_1_), or multiple-exchange tetrads (*E*_n_) [4]. We used the Weinstein method to estimate the frequency of *E*_0_ tetrads for each chromosome in *D*. *virilis*. The X chromosome and third chromosome *E*_0_ tetrad frequencies were estimated at 1.2% and 2.1% respectively. These calculated *E*_0_ tetrad frequencies in *D. virilis* are lower in comparison to *D. melanogaster*, previously estimated to be 5-10% [5,6]. However, the Weinstein method indicated negative *E*_0_ tetrad frequencies ranging from -2.6% to -3.9% for the second, fourth, and fifth chromosomes. Negative values were also sometimes obtained for other exchange class frequencies (Table S10). Negative tetrad frequencies are a drawback to using the classic Weinstein method [5]. Nonetheless, these results indicate that there are many fewer non-exchange tetrads in *D. virilis* compared to *D. melanogaster*, consistent with the higher estimated rate of recombination per Mb in *D. virilis*

**Supplemental References**

1. Housworth EA, Stahl FW. Crossover interference in humans. Am J Hum Genet. 2003;73:188–97.

2. Broman KW, Weber JL. Characterization of human crossover interference. Am J Hum Genet. 2000;66:1911–26.

3. de los Santos T, Hunter N, Lee C, Larkin B, Loidl J, Hollingsworth NM. The Mus81/Mms4 endonuclease acts independently of double-Holliday junction resolution to promote a distinct subset of crossovers during meiosis in budding yeast. Genetics. 2003;164:81–94.

4. Weinstein A. Coincidence of Crossing over in *Drosophila melanogster* (*Ampelophila*). Genetics. 1918;3:135–72.

5. Zwick ME, Cutler DJ, Langley CH. Classic Weinstein: tetrad analysis, genetic variation and achiasmate segregation in *Drosophila* and humans. Genetics. 1999;152:1615–29.

6. Hughes SE, Miller DE, Miller AL, Scott Hawley R. Female meiosis: synapsis, recombination, and segregation in *Drosophila melanogaster*. Genetics. 2018;208:875–908.

**Table S9:** Interference values (*nu*) and frequency of crossovers created in the non-interference pathway (*P*) for all chromosomes. Both values were estimated with the Housworth-Stahl model for the entire dataset.

|  | **Chromosome** | | | | |
| --- | --- | --- | --- | --- | --- |
|  | **X** | **2** | **3** | **4** | **5** |
| *Nu* | 3.22 | 3.17 | 2.68 | 3.09 | 3.37 |
| *P* | 4.81E-02 | 1.05E-02 | 2.73E-08 | 6.91E-03 | 9.75E-03 |

**Table S10:** Tetrad frequencies for each chromosome of the *D. virilis* BC1 progeny. The tetrad frequencies were estimated using the Classic Weinstein method from Weinstein (1918). Negative tetrad frequencies are biologically meaningless and a drawback to using this method.

|  | **N-Exchange (*E_n_*) Tetrad Frequency** | | | | | |
| --- | --- | --- | --- | --- | --- | --- |
| **Chromosome** | ***E*_0_** | ***E*_1_** | ***E*_2_** | ***E*_3_** | ***E*_4_** | ***E*_5_** |
| X | 0.012 | 0.176 | 0.039 | 0.580 | 0.116 | 0.077 |
| 2 | -0.040 | 0.184 | -0.034 | 0.754 | -0.135 | 0.271 |
| 3 | 0.022 | -0.031 | 0.556 | 0.184 | 0.193 | 0.077 |
| 4 | -0.034 | 0.087 | 0.213 | 0.618 | 0.000 | 0.116 |
| 5 | -0.027 | 0.167 | 0.222 | 0.367 | 0.271 | 0.000 |

**Figure S4:** The distribution of total crossover counts in *D. virilis* BC1 progeny. The line is the expected number of crossovers under a Poisson distribution given the mean number of crossovers among all samples.


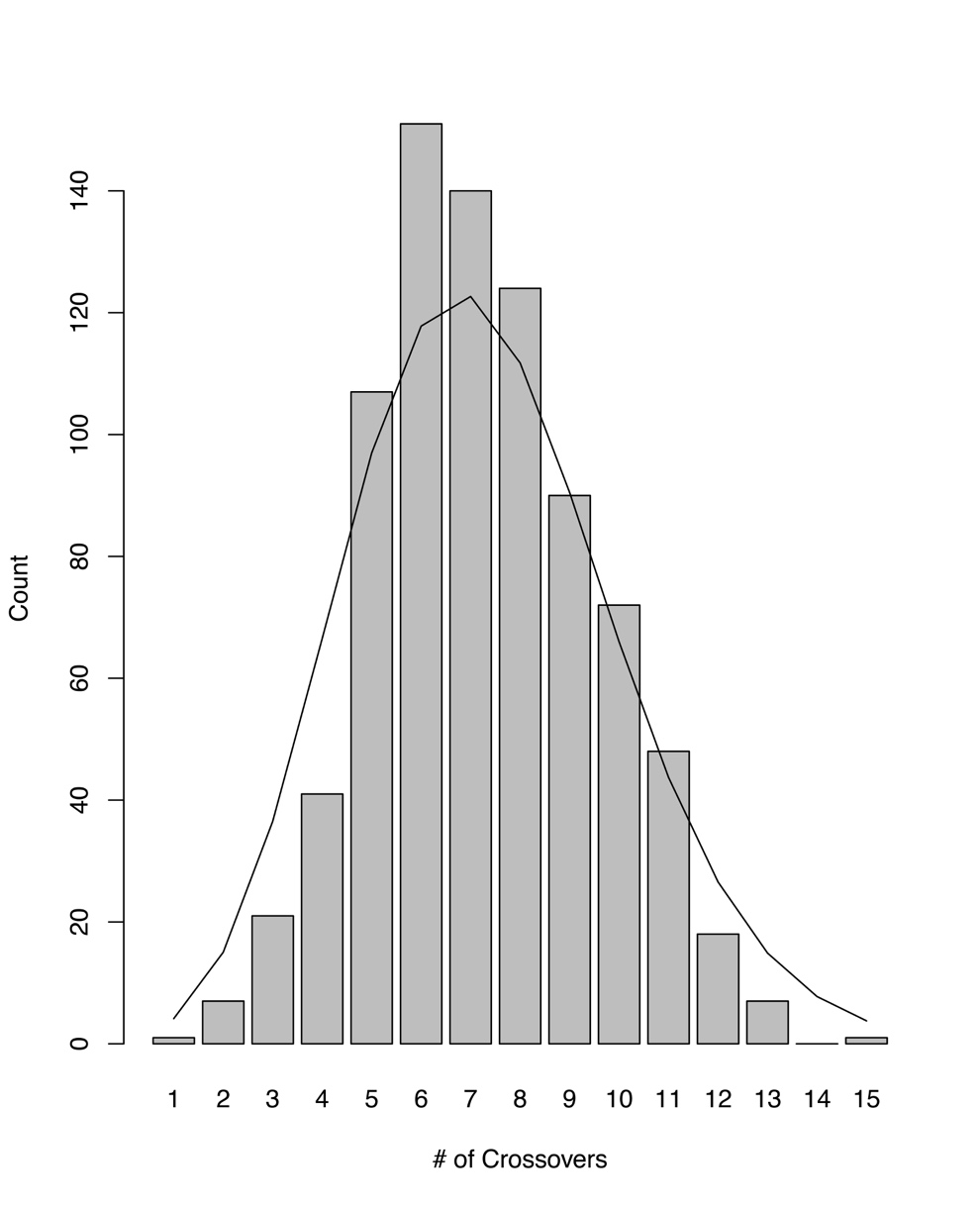

Supplement: Supplementary file 5 — Additional file 5: Interference and tetrad analysis in Drosophila virilis. [file 13100_2020_205_MOESM5_ESM.docx]
